# Selection in a growing colony biases results of mutation accumulation experiments

**DOI:** 10.1038/s41598-022-19928-5

**Published:** 2022-09-14

**Authors:** Anjali Mahilkar, Namratha Raj, Sharvari Kemkar, Supreet Saini

**Affiliations:** Department of Chemical Engineering, Indian Institute of Technology Bombay, Powai, Mumbai, 400076 India

**Keywords:** Evolution, Microbiology

## Abstract

Mutations provide the raw material for natural selection to act. Therefore, understanding the variety and relative frequency of different type of mutations is critical to understanding the nature of genetic diversity in a population. Mutation accumulation (MA) experiments have been used in this context to estimate parameters defining mutation rates, distribution of fitness effects (DFE), and spectrum of mutations. MA experiments can be performed with different effective population sizes. In MA experiments with bacteria, a single founder is grown to a size of a colony (~ 10^8^). It is assumed that natural selection plays a minimal role in dictating the dynamics of colony growth. In this work, we simulate colony growth via a mathematical model, and use our model to mimic an MA experiment. We demonstrate that selection ensures that, in an MA experiment, fraction of all mutations that are beneficial is over-represented by a factor of almost two, and that the distribution of fitness effects of beneficial and deleterious mutations are inaccurately captured in an MA experiment. Given this, the estimate of mutation rates from MA experiments is non-trivial. We then perform an MA experiment with 160 lines of *E. coli*, and show that due to the effect of selection in a growing colony, the size and sector of a colony from which the experiment is propagated impacts the results. Overall, we demonstrate that the results of MA experiments need to be revisited taking into account the action of selection in a growing colony.

## Introduction

Mutations create genetic diversity in a population. The diversity and the relative frequency of beneficial, neutral, and deleterious mutations in a given environment dictate the ability of a population to respond to an adaptive challenge. A direct way of studying this diversity is with the help of a mutation accumulation (MA) experiment. In these experiments, a population is allowed to expand to a fixed size, and forced to go through a severe bottleneck. In this bottleneck, for instance, only one (or a small number of) randomly chosen individual is allowed to progress to the next generation. Since selection is not allowed time to act on this population, the mutations so accumulated are believed to be largely independent of selection, and hence, represent the true spectrum of occurrence of mutations in an organism.

MA experiments have been performed in a number of species^1^, including multicellular eukaryotic species like *Arabidopsis thaliana*^2,3^, *Caenorhabditis elegans*^4,5^, *Drosophila melanogaster*^6–10^. MA experiments have also been performed on single-celled eukaryote *Saccharomyces cerevisiae*^11–14^ and *Chlamydomonas reinhardtii*^15^ and with bacteria such as *Escherichia coli*^16^ and *Salmonella typhimurium*^17^. One of the most consistent patterns from MA experiments has been that as the number of transfers progress, the mean fitness of independent lines decreases, and the variance of the mean fitness between independent lines increases^16^.

MA experiments have provided us with several insights into mutational events of an organism. These include (a) the overall mutation rate^16,18^, (b) the general mutational pattern^13,19–23^, (c) estimating the shape of the distribution of fitness effects of mutations^24–26^. While in eukaryotes, the longer generation times and larger body size permit separation between every generation; the same is not possible in prokaryotes like bacteria, or single-celled eukaryotes like yeast. As a result, mutation accumulation experiments in yeast and bacteria are performed by growing cells for several generations on solid agar. After a period of growth (~ 20–25 generations), a colony is selected randomly and cells from it spread on agar plates (Fig. S1). This process is repeated for a number of transfers (typically ~ 100). In this process, cells with beneficial, deleterious, or neutral mutations lead to formation of a colony. Only cells carrying lethal mutations do not manifest as colonies. Selection of an individual, which forms the colony to be picked is completely randomized, and hence, the effects of selection are thought to be minimized in such an experiment. One of the reasons attributed to this is that since nearly half the cell divisions (and consequently mutations) appear in the last generation, a bottleneck is imposed before selection can act on these mutations^27^. The repeated bottlenecks then drive accumulation of mutations primarily via drift^1^. However, to what extent does action of selection during the growth phase of a colony dictate the outcome of an MA experiment is not clearly known^28^. This aspect of MA experiment has been addressed in literature^29^. For example, using *Pseudomonas* as the model organism, it was demonstrated that large beneficial mutations can impact fitness trajectories in an MA experiment^28^. A recent report has proposed analysis to process fitness data from an MA experiment to remove the effects of selection^30^. Moreover, a number of MA studies have demonstrated that the spectrum of mutations and the mutation rate is a function of the environment in which the organism is propagated^14,31–34^, making results from MA experiments limited to the precise environmental conditions in which the experiment is performed.

In this work, we show that selection plays a significant role in dictating the evolutionary dynamics in an MA experiments with microorganisms. The observed mutational spectrum from the MA experiments does not mimic the underlying distribution from which mutations occur randomly. In fact, as a manifestation of an MA experiment, deleterious mutations are significantly under-represented and beneficial mutations over-represented by a factor of almost two. The distribution of fitness effects of beneficial and deleterious mutations as observed in an MA experiment, and the underlying theoretical distribution are in qualitative agreement, but quantitatively different. Thus, estimates of DFE from an MA experiment are not quantitatively representative of the underlying DFE. In addition, estimates of mutations and the fraction of mutations that are deleterious are likely to be not accurate, when derived from MA experiments. Our MA experiment results, not surprisingly, demonstrate that the effect of selection can be clearly seen when propagation is done from colonies of different sizes, or when cells are propagated from different sectors of a colony. Thus, different sectors of a colony are under different selection pressures. Overall, we demonstrate that inferences about mutational spectrums of bacteria/yeast from mutation experiments are strongly influenced by selection.

## Results

### Increase in fitness of mutation accumulation (MA) experiment lines

We performed MA experiments by propagating *E. coli* (22 lines) on LB plates for a total of 80 transfers. This work started with the observation that many lines of our MA experiment exhibited a growth rate higher than that of the ancestor. Growth rate (proxy for fitness) of the evolved lines at 20, 40, and 80 transfers, is shown in Fig. S2. Somewhat surprisingly, even as the mean growth rate of the 22 lines decreased, 10 out of the 22 lines exhibited an increase in the growth rate by 40 transfers. The number of lines, which exhibited fitness greater than that of the ancestor, decreased to seven at transfer 80. Growth rate of each of the 22 lines at these three time points (20, 40, and 80 transfers) show that of all data points, which exhibited a statistically significant change in growth rate between two successive points of measurement, ~ 45% exhibited an increase in growth rate, and ~ 55% exhibited a decrease. The ratio between the two is not indicative of the ratio of beneficial to deleterious mutations believed to be available to a genotype, as, when compared with available beneficial mutations, available deleterious mutations are thought to be higher in number and on average, stronger in effect^35–37^.

While increase in growth rate in MA experiments has been reported previously^7,38–42^, it has been attributed to action of drift^1^. Repeated bottlenecks in the population, after the period of colony growth (~ 25 generations) are believed to largely negate the effect of selection. However, this idea remains based largely on qualitative intuition. This result formed the basis for our simulation and experiments, as described in this work.

### Selection in action during growth in a colony

**Figure 1:**
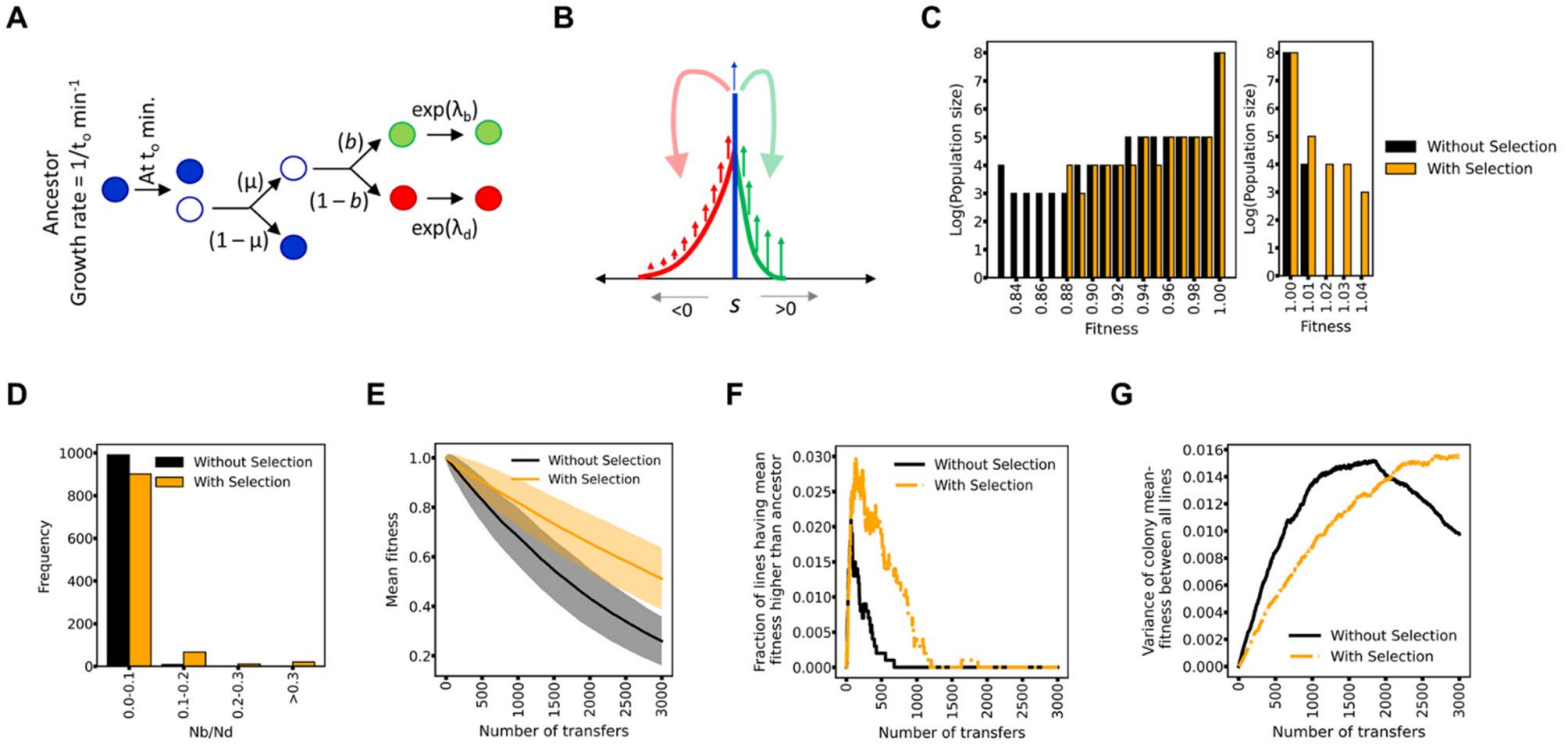
(**A**) Cell division model. At time 0, a cell (with growth rate 1/*to*) was assumed to divide into two. The progeny acquires mutation with probability μ. he acquired mutation is beneficial with a probability *b*. The selection coefficient of a mutation was drawn randomly from an exponential distribution with parameters *λ*_*b*_ and *λ*_*d*_, for beneficial and deleterious mutations, respectively. (**B**) Colony growth model. After growth to a size of 60,000 individuals as described in (**A**), the growth of individuals of genotype was modeled deterministically. Red (green) indicates the individuals with deleterious (beneficial) mutations. Blue indicates individuals with no mutations. As the colony size grows, in each generation, newer mutations occur in the parent genotype, and this contributes to the pool of beneficial and deleterious mutants. The number of such mutants is computed simply as *Nμ*, where *N* is the number of individuals of ancestral genotype in that generation. The fitness effects of such mutants were allotted as per the exponential distribution describing beneficial and deleterious mutations. This process is continued till the colony size reaches a fixed number. (**C**) Frequency distribution of individual cells in a single colony for genotypes with deleterious (left) and beneficial (right) mutations. The two distributions (with and without selection) are statistically different from each other, for both deleterious (*p* value 0.09) and beneficial mutations (*p* value 0.01) (Kolmogorov–Smirnov test) (**D**) Frequency distribution of the ratio of the number of individuals carrying beneficial mutations (*N*_*b*_) and the number of individuals carrying deleterious mutations (*N*_*d*_) in a single colony. 1000 colonies were simulated, and the frequency distribution is represented. (p < 10^–8^, Kolmogorov–Smirnov test) (**E**) Mean fitness (solid line) in an MA experiment (for 2000 transfers) and the standard deviation between the fitness of individual lines (shaded region). (Obtained *p* values < 0.05 by applying unpaired t-test at population before each transfer point) (**F**) Fraction of the lines with fitness greater than that of the ancestor, as a function of number of transfers. (**G**) Fitness variance between lines as a function of number of transfers (*p* values < 0.05 Levene test at population before each transfer point, except at the intersection of curves). All simulations were performed for *K* 6 × 10^8^, colony size 10^8^, and *b* 0.05.

In this section, we describe results of computer simulations of MA experiments. Starting with an ancestor with fitness (1/*t*_*o*_) (where *t*_*o*_ is the division time), growth of a colony is mimicked as shown in Fig. 1A and B (see “Methods” for more details). The relevant parameters for the simulations are listed in Table S1. In Fig. 1C, the frequency distribution of cells with different fitness is shown for a single colony, when in-colony selection acts (“with selection”). As shown, the frequency distribution is qualitatively different from the control simulation in which it was assumed that fitness effects of mutations do not change growth rate in a colony environment—i.e. all cells grow at the same rate (when in-colony selection is absent, “without selection”).

However, the process of mutations is inherently stochastic. Effects like the timing and nature of mutations deeply impact the final genetic structure of the colony. Thus, the number of beneficial and deleterious mutants present in a colony vary significantly from one colony to another (Fig. 1D). The distribution of the ratio *N*_*b*_/*N*_*d*_ (ratio of the number of individuals with a beneficial mutation and the number of individuals with a deleterious mutations) across a simulation of 1000 colonies exhibits a wide distribution. This distribution is significantly different from the distribution of *N*_*b*_/*N*_*d*_ expected if selection did not act in a growing colony. The null expectation of this ratio, in the absence of selection is ~ 0.05. However, due to selection, this ratio is increased by approximately 20%. Thus, selection acting in a growing colony qualitatively changes the distribution of the number of individuals carrying beneficial and deleterious mutations.

### Mimicking an MA experiment

From the simulation of a colony, we extend our simulations to mimic an MA experiment. Starting from an ancestor, we simulate colony growth to a given size. Upon reaching the appropriate colony size, the individuals are binned in fitness levels. From this distribution, we pick an individual randomly, and similarly simulate the kinetics of colony growth again. The likelihood of picking a particular fitness phenotype as a founder is simply proportional to the frequency of the genotype with the corresponding fitness. By repeating this process, we mimic an MA experiment.

The fitness of an MA line decreases with time. In Fig. 1E, the mean fitness and the standard deviation of 1000 independent lines is shown. The qualitative trajectory of the mean fitness with the number of transfers is consistent with previous experimental observations^16^. The mean fitness trajectory is statistically significantly different from the trajectory obtained with the assumption that selection was absent during growth in a colony.

A large fraction of MA lines exhibit an increase in fitness, in the early parts of the simulation. The fraction of MA lines with increased fitness exhibits a characteristic pattern, and the variance of fitness among MA lines increases with the number of transfers (Fig. 1F and G). We note that as an MA experiment proceeds, the fraction of lines with fitness less than the ancestor decrease. We note that during the course of an MA experiment, these lines also acquire (and continue to acquire) beneficial mutations. Their lower fitness is simply due to a larger number of deleterious mutations. As a result of the action of selection, the fraction of lines with fitness greater than the ancestor is qualitatively higher in the experimental simulation as compared to the control simulation (Fig. 1F). This action of selection was also found to homogenize behavior between lines, as compared to the control, as shown in the variance of mean fitness between independent MA lines (Fig. 1G). However, after a certain number of transfers, because different trajectories following unique adaptive paths, the variance of fitness is higher in the case when selection acts (also see Fig. S3). (Also see Fig. S4, when fitness is plotted against number of doublings, rather than number of transfers).

### DFE from an MA experiment exhibits bias towards beneficial mutations

From our simulation results, we next investigate the (a) DFE and (b) mutation rate estimates from an MA experiment data. We first discuss (a). DFE of beneficial and deleterious mutations are recorded from the simulations via two parameters. First, of all the mutations occurring in the MA simulation, what fraction are beneficial. Second, what are the corresponding DFEs for beneficial and deleterious mutations. For both these parameters, we extract observations from the MA simulations and compare with the underlying parameters/distributions, which formed the basis of our MA simulations.

The action of selection biases MA experiments towards a greater observation of beneficial mutations. Depending on the colony size, the percent of beneficial mutations increase from ranging from ~ 20 to ~ 33%. The number of excess beneficial mutations increase with increasing colony size (Fig. S5).

**Figure 2:**
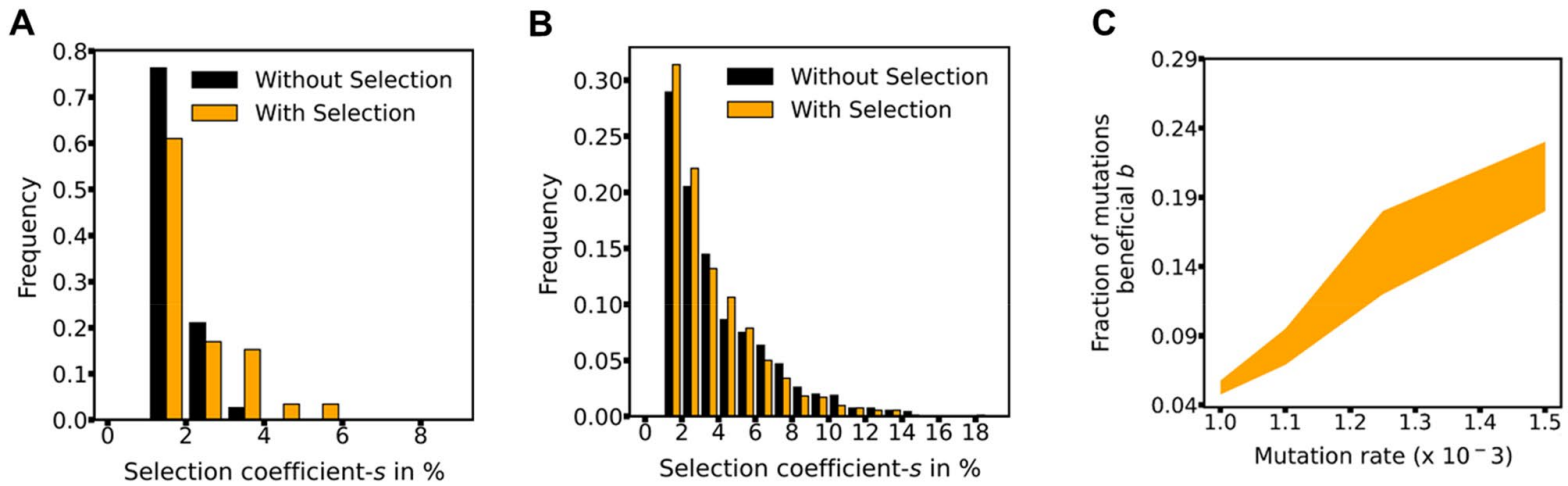
DFE estimates from an MA experiment are biased by selection. (**A**) Frequency distribution of beneficial mutations in an MA experiment. The observed distribution is best explained by λ 1.26 (95% CI = [1.18, 1.34]). The control distribution is explained by λ equal to 1.88 (95% CI = [1.76, 2.0]). The underlying theoretical distribution was λ equal to 1. (**B**) Frequency distribution of deleterious mutations in an MA experiment. The observed distribution is best explained by λ 3.13(95% CI = [2.90, 3.33]). The control distribution is explained by λ equal to 3.50 (95% CI = [3.25, 3.70]). The underlying theoretical distribution was λ equal to 3. (**C**) Estimates of *μ* and *b* which explain the observed kinetics of the MA experiment. All simulations were performed for *K* 6 × 10^8^, colony size 10^8^, and *b* 0.05.

For both, beneficial and deleterious mutations, the observed distributions from the MA experiment qualitatively mimic the underlying distributions from which mutations are drawn (Fig. 2A and B). The exponential fit for the beneficial mutations (Fig. 2A) is quantitatively less statistically robust, as compared to that for deleterious mutations (Fig. 2B). In fact, the results from our simulations indicate that MA experiments are likely to not yield quantitatively accurate estimates of DFE of beneficial mutations. However, DFE of deleterious mutations can be estimated to a greater accuracy from MA experiments.

Therefore, a set of parameters define the outcome of an MA experiment. These parameters include (a) mutation rate, *μ*, (b) fraction of mutations which are beneficial, *b*, (c) parameter *λ*_*b*_ describing distribution of beneficial mutations, and (d) parameter *λ*_*d*_ describing distribution of deleterious mutations. This parameter space constitutes a 4-dimensional space, and the challenge is to identify regions of this space, which describes the observations from an MA experiment. We fix *λ*_*b*_ and *λ*_*d*_, and scan the two-dimensional space described by mutation rate *μ*, and fraction of mutations that are beneficial, *b* to fit the data from the MA experiment simulation. As shown in Fig. 2C, the estimate of mutation rate *μ* from the MA experiment simulation is dependent on the parameter *b*. With increasing *b*, higher mutation rates are able to explain the observed MA line trajectory, as observed in the simulation. Since *b* is largely unknown for a particular environment, estimating *μ* from an MA experiment is non-trivial.

### Different MA experimental designs are affected differently by selection

In the above section, we demonstrate that selection, under a given set of parameters, affects MA experiments. The above simulations are done with the parameters, *K*, population capacity of a colony (6 × 10^8^), colony size (the CFU at the time of transfer) (10^8^), and *b* (fraction of mutations which are beneficial) (0.05). Here, we study how does selection shape the outcomes of an MA experiment, as we vary these three parameters.

**Figure 3:**
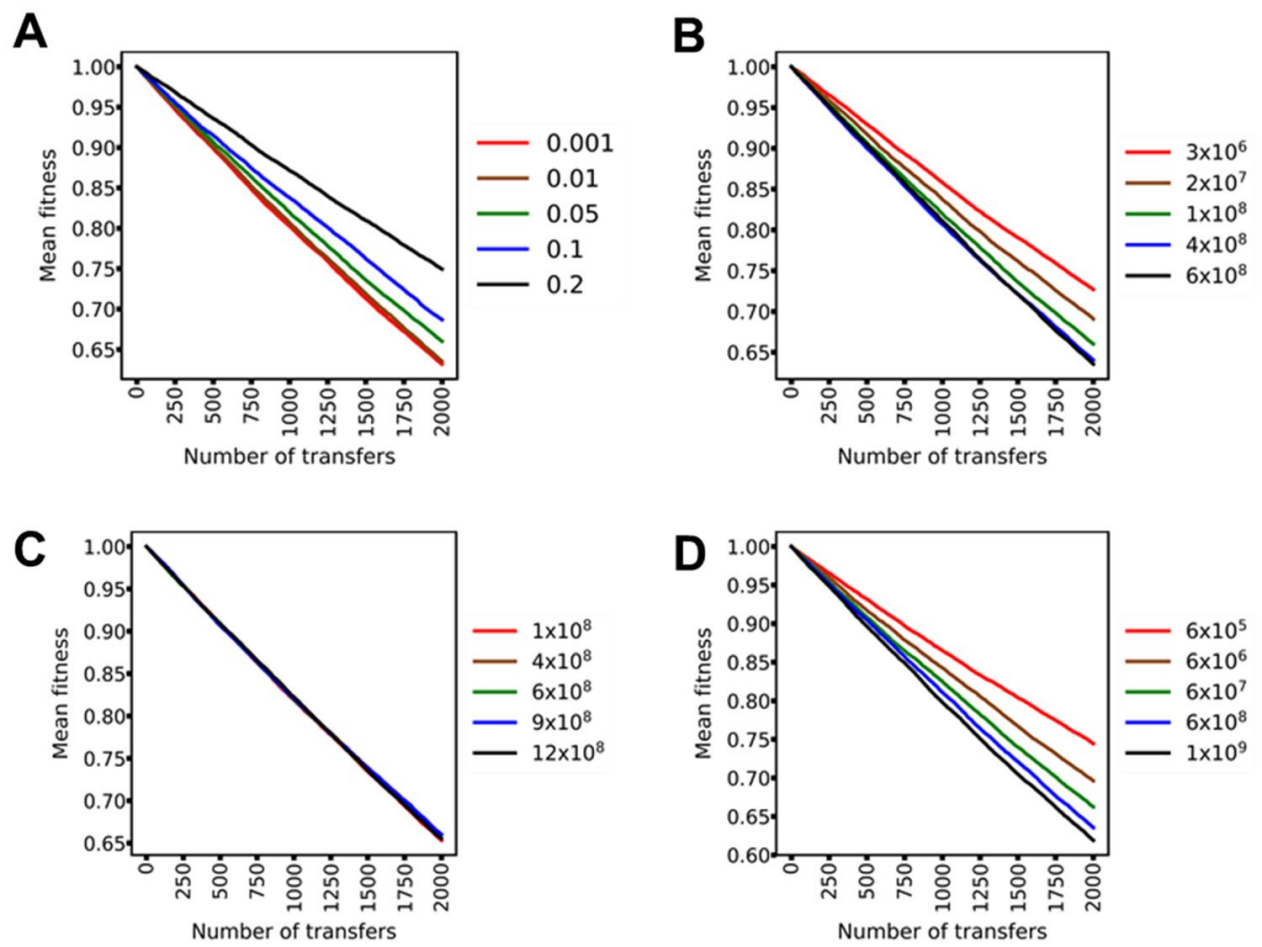
Fitness trajectories of an MA experiment are biased by selection. (**A**) For a given maximum population size (K 6 × 10^8^) and colony transfer size (10^8^), the fraction of mutations which are beneficial (*b*) dictate (legend) the fitness trajectory in an MA experiment. (**B**) For K 6 × 10^8^ and *b* 0.05, the size of the colony at the time of transfer (legend) dictates the fitness trajectory in an MA experiment. (**C**) When for different *K* values (legend) and *b* equal to 0.05, transfer is done at the same colony size (10^8^), MA fitness trajectories are similar to each other. (**D**) The carrying capacity (legend) influences the MA fitness trajectory. In these simulations, *b* equals 0.05, and colony is transferred when its size equals *K.*

Keeping *K* and colony size the same as above, we first mimic MA experiments in our simulations for different values of *b.* As shown in Fig. 3A, the mean rate of decrease of fitness in an MA experiment decreases with increasing *b* (the slope increases linearly with increasing *b*) (Fig. S6). This is accompanied by a greater number of lines exhibiting mean fitness greater than that of the ancestor. The distribution of beneficial and deleterious mutations, however, remains qualitatively the same as the underlying distribution.

We also find that the timing of transfer also dictates the outcomes of the MA experiment. For a given *K* (6 × 10^8^) and *b* (0.05), fitness decrease is the smallest if the transfer is done at small colony sizes (Figs. 3B and S7).

Different environments support colonies of different sizes (Fig. S8). If the transfer is done at the same colony size in any environment (each of which supports a different *K*), the outcomes of the MA experiment are not varied (Figs. 3C and S9). However, if the MA experiment is designed in a fashion such that the colony transfer takes place once the population size reaches *K*, the results of the MA experiment are affected differently by selection (Figs. 3D and S10).

In addition, the mutation rate of the organism plays an important role in dictating the fitness trajectory during an MA experiment. Our simulations show that increasing mutation rates lead to a faster decrease in the mean fitness of a colony when studied as a function of the number of transfers (Fig. S11). This is also reflected in the fraction of lines which have a fitness level greater than that of the ancestor, when tracked with the number of transfers.

In the above analysis, we report fitness data as a function of number of transfers. However, depending on the design of the simulation, the number of cell divisions occurring in each simulation is different from other. We note that if the fitness data were to be plotted against the number of cell divisions, the differences in fitness trajectories between the different simulation schemes (e.g., when transfers are done at different colony sizes) are eliminated (Fig. S12). However, fitness trajectories when studied as a function of number of doublings are different, when the variable under consideration is representative of cellular functioning (like, mutation rate, or, fraction of all mutations that are beneficial).

Many studies report that distribution of beneficial mutations can be approximated by an exponential distribution. However, distribution of deleterious mutations is more contentious^24,43–45^. To test how the above results change if the deleterious mutations are represented by other distributions, we perform simulations where the deleterious mutations are distributed normally, uniformly, or a mixture of normal distributions (Fig. S3). As shown in Figs. S14–16, the choice of deleterious mutation distribution does not alter our results. All three choices of deleterious mutation distributions exhibit (a) an increased number of beneficial mutants in the colony (b) increased sampling of the beneficial mutations, (c) slower decrease in the mean fitness with increasing transfers, compared to the control, and (d) a greater fraction of lines exhibiting increased fitness. Moreover, this aspect of changes in the fitness trajectories is also observed when both, deleterious and beneficial trajectories are represented as combinations of normal distributions (Fig. S17).

### Demonstrating selection in an MA experiment

To demonstrate the action of selection, an MA experiment was performed with 160 parallel lines of *E. coli*, propagated on LB media. The lines were separated into five categories, with 32-identically treated lines in each. The first three categories were MA lines propagated at different colony sizes. These lines are called small (lines s1–s32) (8 h of growth), medium (m1–m32) (12 h of growth), and large (l1–l32) (24 h of growth). These durations of growth correspond to a large change in the CFU count in a growing colony (Fig. S18). In all three lines, an entire colony was picked, resuspended in PBS buffer, and then transferred to the next plate. The other two categories were large colonies where the cells propagated from one transfer to the next were picked from edge of the colony (lines e1–e32) and centre of the colony (lines c1–c32) (Fig. S19).

**Figure 4:**
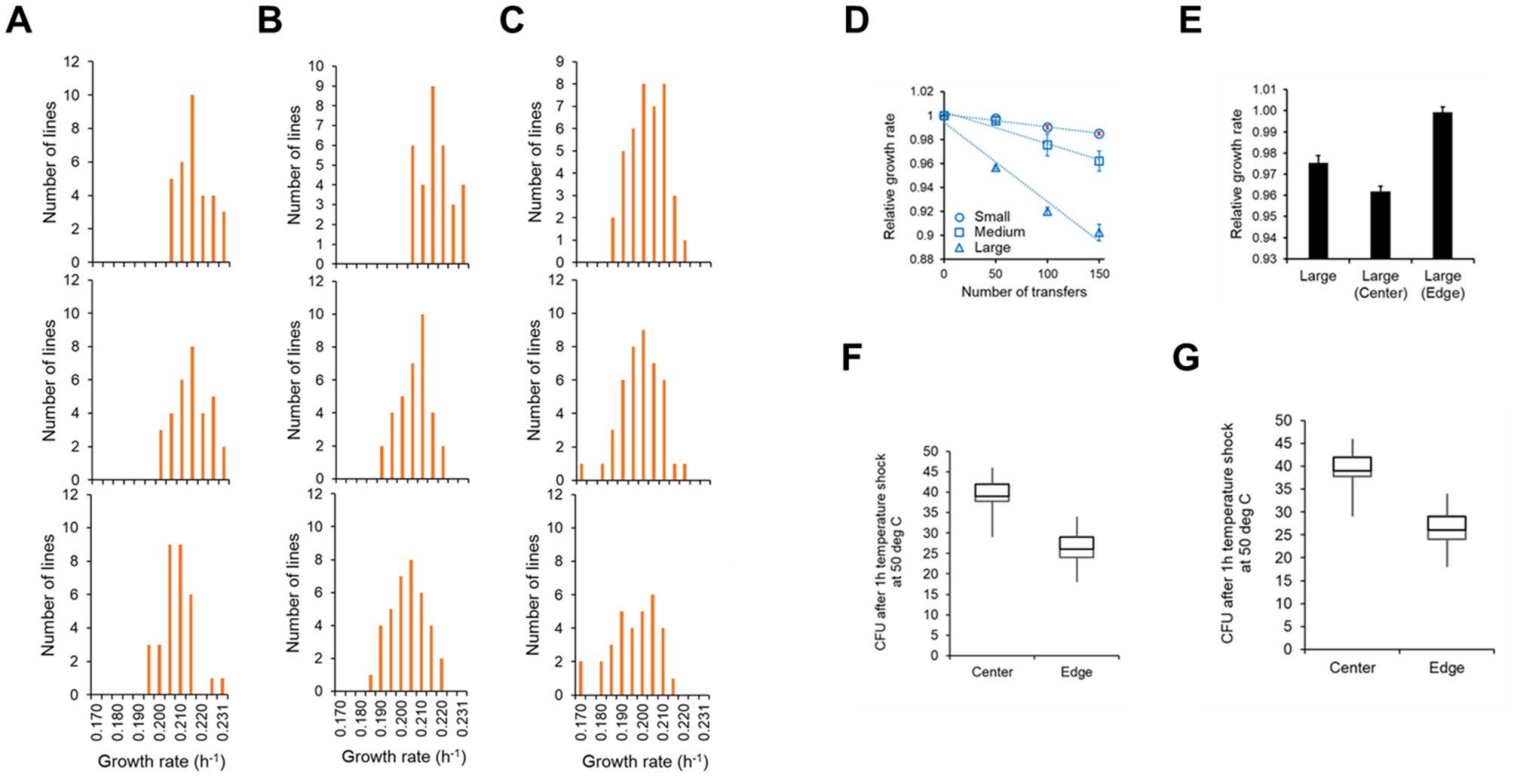
Distribution of fitness of the 32 MA lines for (**A**) small colonies (s1-s32), (**B**) medium colonies (m1-m32), and (**C**) large colonies (l1-l32). In (**A**)–(**C**), op panel represents the fitness distribution after 50 transfers, the middle panel after a 100 transfers, and the lower panel after 150 transfers. (**D**) The mean fitness of the small (s1-s32), medium (m1-m32), and large (l1-l32) lines decreases with the number of transfers. (**E**) Among the 96 lines propagated at large colony size, the 32 lines of large (edge) set exhibit the maximum growth rate, and the 32 lines comprising the large (center) set show the minimum growth rate. (**F**) CFU after thermal stress experiment. (*p* value < 0.00001) (**G**) CFU after solvent stress experiment. (*p* value < 10^–15^) The mean OD of the centre lines is greater than that of the edge lines (t-test, *p* value < 0.0001).

The distribution of fitness of the small, medium, and large lines at transfers 50, 100, and 150 is shown in Fig. 4A–C. The mean fitness of these lines decreases as the number of propagations increase (Fig. 4D). At transfer 50, the difference between the mean fitness of small and large lines is statistically insignificant (*p* > 0.24). All other pairwise comparisons of mean fitness between small, medium, and large lines at 50, 100, and 150 transfers shows a statistically significant difference (*p* < 0.001, unpaired two-tailed t-test). For the three sets of lines propagated at different colony sizes, the rate of decrease of mean fitness is the greatest in the lines of large size, followed by that in the experiment with medium-sized colony. The rate of decrease of fitness was the least in the experiment with the small-sized colony. Although the linear decrease in fitness has been previously reported in MA experiments with bacteria, its variation with colony size at the time of transfer has not been studied before. As we mention in the simulations, this difference in the rate of decrease of fitness is due to fewer mutations in the experiment with small sized colonies. As a result, the ancestral genotype persists for a longer time in the MA experiment, and mean fitness changes significantly slower. While the MA experiments were carried on a plate, and cells grew in a colony environment; fitness values were measured in liquid LB. This means that our results will be biased by pleiotropic effects of mutations^37^.

We explicitly test the assumption that different sectors of a growing colony are under different selection pressures. The microenvironments of a growing colony are known to be quite distinct from each other, and hence, the individuals in these sectors are likely under different selection pressures^46,47^. For example, the selection at the growing edge of the colony is for fastest growing individuals; while the microenvironment at the centre of the colony comprises of environmental (solvent) and nutritional stresses. Continuous propagation of MA lines, while selecting cells from the edge or centre of a colony are likely to yield different phenomenology. We explicitly test this hypothesis.

After 150 transfers, the mean growth rates of the three large lines (l1–l32), (c1–c32), and (e1–e32) were compared. The mean growth rate of the (e1–e32) lines were the largest and that of the c1–c32 lines were the least, and that of the lines l1–l32 between the two (Fig. 4E). This demonstrates the action of selection in the growing colony.

Studies of cellular physiology have demonstrated that a strong relationship between growth rate and stress response exists^48–50^. To test this, when the cells from the lines (edge and center) were subjected to (a) heat shock for one hour or (b) solvent stress for 10 min (see “Methods” for more details), the survival rate for the cells from centre lines (c1–c32) was found to be higher than that from the edge lines (e1–e32) (Fig. 4F and G). Our results thus demonstrate that the microenvironment in a growing colony dictates the evolutionary trajectory of a population. These results provide direct evidence of action of selection during an MA experiment using a microbial system.

## Discussion

In this work, we address the question of how selection impacts the results from an MA experiment. We demonstrate that, during a mutation accumulation experiment with bacteria or haploid yeast, selection acts to significantly bias the nature of the mutations that fix in the population^29^. We show that the assumptions that selection in the colony is minimal since most cell divisions take place in the last generation^27^ or that repeated bottlenecks in an MA experiment makes selection redundant^1^, are not valid during the growth phase of the colony. In fact, selection acts quite strongly in an MA experiment. On average, beneficial mutations are significantly over-represented than expected in the absence of selection. In the context of growth of a colony, starting from a single colony to a population size of ~ 10^7^ constitutes ~ 25 generations. The effective population size of the population in this phase is ~ 10^51^, and selection acts on this fluctuating population size.

As shown in our results, the estimates of mutation rates, DFEs, trends of mutation rates obtained from MA experiments are biased by selection. The manifested mutations are a result of selection and chance, and this process is dictated by several parameters, which feed into our model. These factors include mutation rate, fraction of mutations that are beneficial, DFE of beneficial and deleterious. The problem of deciphering mutation rate (or other parameters) from phenotypic or genotypic data from an MA experiment then becomes an inverse problem, such as one attempted previously in the context of DFE of beneficial mutations^52^. In this work, the authors attempted to obtain the DFE from a distribution of beneficial mutations that escaped drift. However, multiplicity of the solution meant that DFE could not be estimated through this strategy.

MA experiments are thought to be independent of bias of selection since most of the mutations are thought to take place in the last generation of colony growth. As a result, selection does not get a chance to act on these mutations. However, colonies where mutations occur early are not free from the action of selection, and they significantly alter the growth kinetics of beneficial and deleterious mutants. Moreover, colonies have spatial structure, and the role of selection can play out in a number of ways in the three-dimensional structure of a colony^53^. The role of expansion in dictating population structures and evolutionary trajectories has been recently studied in the context of biofilms^54^, and colonies^55,56^.

Our work demonstrates that selection impacts an MA experiment in a number of ways. The dynamics of fitness of an MA line is dependent on not only the size of the colony (which is dependent on the environment in which the MA experiment is being performed) but also on the timing of propagation of cells from one colony to the next plate. Since the mutational spectrum that is beneficial in one environment is known to be quite different from another environment^31–34^, MA experiments performed in different environments exhibit unique mutational signatures.

Two factors dictate the relative strengths of selection and drift acting on a colony. As the colony grows to a greater size, selection has greater time to act on the existing genotypes in the colony (particularly those mutations that arose in the first few generations). On the other hand, greater colony size implies that a large number of divisions (and hence mutations) take place in the last generation—and hence, selection cannot act on the genetic diversity so generated. This is important since the size of the colony is dictated by the media on which the MA lines are propagated. Our simulations predict that the slope of the mean fitness trajectory exhibits a monotonic, decreasing relationship with the size of a colony at the time of transfer. At small colony sizes, the mean time for an individual carrying a mutation to be propagated in an MA experiment is considerably longer than that in an experiment with larger colony size. As a result, the rate of decrease of fitness in a smaller colony is significantly smaller than in a larger colony.

Moreover, a colony is a highly heterogeneous environment, which leads to different selection pressures and phenotypic manifestations of the participating individuals^57–60^. The fitness trajectories of an MA experiment, when propagated from the edge of a colony would be quite different from an MA experiment where propagation is done from the center of the colony. For instance, in yeast and bacteria, local environments within a colony are known to elicit different physiological responses^61–63^. Similarly, the size of the colony at which propagation is done is also an important parameter. CFU increase in a growing colony is initially exponential with time, and then slows down to zero. This implies that, in addition to different selection pressure in different regions of a colony, the relative strengths of drift and selection are also changing with time in a growing colony.

MA experiments have been used to estimate mutation rates^16,17^. However, these studies assume that beneficial mutations are sufficiently rare that they are not picked up due to selection in MA experiments. In addition, mutations with large deleterious effects (lethal mutations) can be ignored from the analysis. Hence the assumption that it is deleterious mutations only, which are relevant in the context of MA experiments. However, recent work has shown that the fraction of all mutations that are beneficial can be significant^26,64–67^.

## Methods

### Simulations

#### Model assumptions

While modelling cell growth in a colony, we make the following assumptions.

1. It is known that division times of isogenic populations are represented by log-freschet distributions^68^. In this scheme, all isogenic individuals are assumed to divide at the exact time. This assumption was relaxed when the division time for an individual from an isogenic population was chosen randomly from a normal distribution with a standard deviation of five percent around the mean. Experimental data sets from studies using microfluidic devices suggests that the coefficient of variation of division time is 5–10% of the mean division time in isogenic populations^69–71^.
2. Recent work on microbial systems has demonstrated that epistasis influences the dynamics of adaptation^72–74^, and several aspects of this epistasis are known^75–78^. We, however, do not include the effect of epistasis in our model, and assume additive effect of mutations on fitness.
3. Effect of neutral mutations. The effect of a mutation on the fitness of an organism can be beneficial, deleterious, or neutral. While neutral mutations do not change the fitness of the individual, they have the potential of changing the adaptive trajectory of the organism^79–82^. In this context, it has been suggested that an intermediate fraction of all mutations being neutral can speed up the adaptive response of a population, and can thus be under secondary selection^79^. We do not consider this effect in our model.
4. We assume that mutational supply is very large, compared to the duration of the experiment. Hence, the DFE does not change with the changing genotype.

### Simulation of colony growth

The growth of a colony is simulated starting from a single haploid individual. The growth of a colony is divided into two phases. In the first, starting from the founder individual, growth is modelled stochastically up to a size of 60,000 individuals. This phase is followed by a deterministic phase of growth, up to the final colony size.

The stochastic phase is simulated as follows. The cell division time of the founder of the colony is assumed to be 100 min (*t*_*o*_). When cell division occurs, a progeny identical to the parent is generated with a probability (1 − *μ*). The progeny has a mutation with probability *μ*. The acquired mutation is beneficial with probability *b*, and deleterious with probability (1 − *b*). Neutral mutations are not taken into account in our model. It is, however, known that neutral mutations can influence the evolutionary trajectories of population^79,80,82^. An exponential distribution is assumed for both beneficial and deleterious mutations. The distributions are represented by parameters *λ*_*b*_ and *λ*_*d*_ for beneficial and deleterious mutations, respectively. It has, however, been proposed that distributions describing deleterious mutations are more complex^43,45,83–85^. The parameters for the simulations are given in Table S1.

Once an individual is simulated to have acquired a beneficial mutation, the magnitude of the beneficial mutation is identified by a random draw from the exponential distribution with parameter *λ*_*b*_. The same strategy is followed for deleterious mutations. After acquisition of a mutation, the division time of the progeny is updated accordingly.

In this phase of growth, the individual closest to its time of division is identified and chosen for division. Imagine a population size of three (bacteria A, B, C). A divides every 100 min, B every 96 min, and C every 110 min. Thus, if these three individuals have just resulted from a cell division, they are 100, 96, and 110 min from the next cell division, respectively. In such a scenario, individual B is closes to its time of division. For all other cells, the time to division is updated accordingly. This process is continued till the population reaches a size of 60,000.

After growth of the population to a size of 60,000, further expansion to a full colony size is modelled via a deterministic scheme (see Fig. S17 for empirical determination of full size of a colony). The genotypes in the colony at size 60,000 are recorded and distributed in bins of discrete fitness values. Further growth is modelled in steps, where each step is one generation time of the founder genotype. In this step, newer mutations arise in all genotypes in the growing colony. The new mutants from each bin are placed in appropriate bins, after determination of their fitness as per the underlying DFE. For example, if the number of individuals in a particular fitness bin is *P* at a certain time step. In the next generation, *P*(1 − *μ*) identical individuals are added to the number of individuals of this genotype. *Pμb* individuals with beneficial (with respect to this bin) mutations are added to the population; and *Pμ*(1 − *b*) individuals with deleterious (with respect to this bin) mutations are added to the population. The fitness values of the mutants in this round of growth is allotted as per the exponential distributions defined by *λ*_*b*_ and *λ*_*d*_. This process is continued till the population of the colony reaches a particular size, as described in each simulation*.*

Individuals in each bin, are assumed to grow with a growth rate equal to the average fitness of the bin. A set of coupled ODEs of the form (1) are solved numerically till the population size reaches colony size, using ode45 on MATLAB.

1 $$ \frac{{dN_{i} }}{dt} = r_{i} N_{i} \left( {1 - \frac{{\mathop \sum \nolimits_{i = 1}^{B} N_{i} }}{K}} \right) $$

where *K* is the maximum colony size in a particular environment. In this work, the size at which the transfer takes place is referred to as colony size. The colony size is less than or equal to the K, for any given environment. In this work, *K* is taken to be 6 × 10^8^. Simulations for different colony sizes are performed by changing the value of colony size, *b,* or *K*. The process is summarized in Fig. 1B.

### Simulating MA trajectory

Colony growth, using the above described simulation scheme, was simulated for MA experiments. After growth of a colony to size *K*, one individual was picked randomly from the colony. This individual was then considered as the founder for the next transfer in an MA experiment. This process was continued for 2000 transfers. In simulations where the effect of selection was ignored. The acquisition of mutations and the distribution of fitness across members of a colony was recorded. However, it was assumed that these mutations did not change the phenotype of the individual cells, and each cell divided at the same rate as that of the ancestor.

### Calculation of *b* and μ pairs which explain the MA fitness trajectory data

For the MA simulation for K (maximum CFU carrying capacity of a plate environment) 6 × 10^8^, colony size (CFU size at which transfer takes place in an MA experiment) 10^8^, and *b* (fraction of mutations which are beneficial) equal to 0.05, the mean fitness of colonies of all thousand lines after every transfer step was calculated. The 95% confidence interval around each of the mean fitness value obtained after every transfer step was then determined. MA simulations were performed with different μ and *b* to determine the window of these parameters which give MA trajectories within the 95% confidence interval.

### Estimates of DFE parameters

Estimate of the DFE for beneficial and deleterious mutations were done by fitting a *λ* parameter on the observed distribution of first mutations in simulated MA experiments. The fitted parameters were then compared with the underlying theoretical distribution (*λ* in Table S1). To perform this comparison, we fit an exponential function for the obtained sample of fitness effects and predicted the mean of the underlying distribution using maximum likelihood estimator (Python’s scipy satistics library).

## Experiments

### Media and reagents

Luria–Bertani (LB) media was used for all experiments.

### Mutation accumulation experiment

*E. coli* K12 MG1655 (ATCC 47076) was revived from freezer stock in LB and grown overnight at 37 °C and 250 rpm. A single colony was used as a founder for all mutation accumulation lines. At every transfer, prior to spreading the cells on the plate, an area was marked. The colony in (or closest to) the marked area was chosen for the next transfer for each line.

Twenty-two independent lines were carried forward in the manner described above for the data presented in Fig. S1.

For the experiment corresponding to Fig. 4 (and the associated data in the Supplement): thirty-two independent lines were propagated for each of the following five conditions: (a) transfer after 8 h of growth on LB plates at 37 °C. These 32 lines are referred to as “small1” (s1) to “small32” (s32); (b) transfer after 12 h of growth on LB plates at 37 °C. These 32 lines are referred to as “medium1” (m1) to “medium32” (m32); (c) transfer after 24 h of growth on LB plates at 37 °C. These 32 lines are referred to as “large1” (l1) to “large32” (l32); (d) transfer after 24 h of growth at 37 °C on LB plates, where only cells from the outermost edge of the selected colony are propagated further. These 32 lines are referred to as “edge1” (e1) to “edge32” (e32); and (e) transfer after 24 h of growth on LB plates at 37 °C, where cells from the center of the colony are propagated further. These 32 lines are referred to as “center1” (c1) to “center32” (c32). The colony size and areas picked for propagation are as shown in Fig. S16. For the “edge” and “center” experiments, to determine the number of cells picked from a colony, the part of the colony picked was suspended in PBS and appropriate dilutions plated on LB plates. The CFU on the LB plates were counted to estimate the number of cells picked. Approximately 10^8^ cells were picked from a colony in the “edge” and “center” experiments.

The MA evolution experiment was carried out for a total of 150 transfer for each of the five groups of 32 lines. The evolved lines were stored after every 50 transfers at − 80 °C in 25% glycerol.

### Growth rate estimation of MA lines

For calculating the relative fitness of the MA lines, cells from the MA lines and the ancestor were revived from the freezer stock in 2 ml LB. The cultures were then grown for 12 h at 37 °C and 250 rpm. The cultures were then sub-cultured in fresh LB media to an initial OD600 of 0.01. A volume of 150 μL of these cultures were transferred to a 96-well clear flat-bottom microplate (Costar) in triplicates. The cultures were grown at 37 °C in a microplate reader (Tecan Infinite M200 Pro), until they reached stationary phase. OD600 readings were taken every 30 min with 10 min of orbital shaking at 5 mm amplitude before the readings. A gas permeable *Breathe-Easy* (Sigma-Aldrich) sealing membrane was used to seal the 96-well plates. Growth rates were calculated as described in^26^.

### Temperature stress experiment

Cells were revived from the freezer stock in 2 ml LB and allowed to grow at 37 °C for 12 h. The cultures were then diluted in 2 ml water to an OD600 of 0.2, and immersed in a hot water bath at temperature 50 °C for a duration one hour. After the heat shock, 100 μl of 1:100 dilution of the cultures were spread on LB plates for single colonies. The number of CFU for each line was recorded.

### Solvent stress experiment

Cells were revived from the freezer stock in 2 ml LB and allowed to grow at 37 °C for 12 h. The cultures were then diluted in 2 ml water containing 10% (v/v) ethanol (Honeywell, Catalog No. 32221) to an OD600 of 0.2. The diluted cultures were kept at 37 °C for 10 min, with shaking at 250 rpm. After the solvent shock, 100 μl of a 1:100 dilution of the cultures was spread on LB plates for single colonies. The number of CFU for each line was recorded after 18 h of growth on the plates.

### Plate and colony images

Representative images of colonies after 8, 12, and 24 h of growth on LB plates at 37 °C were taken as follows. Ancestor *E. coli* was streaked from a glycerol freezer stock onto an LB plate and incubated at 37 °C for 12 h. A single colony from the plate was restreaked onto a fresh LB petri plate (90 mm, polystyrene) and incubated at 37 °C. Plate images were taken at 8, 12 and 24 h of incubation at 37 °C in UVITECH gel documentation unit in white light at an exposure of 280 ms, 4 × zoom and 540 focus using the Essential V6 software. The background color was inverted for clarity and zoomed colony images were obtained using FIJI^86^.

### CFU count in a colony

*E. coli* K12 MG1655 culture was started from a single colony. Cells were grown at 37 °C with 250 rpm for 24 h. Cells were then diluted and plated for single colony on the following three media (a) LB, (b) 0.5% glucose (M9 salts), and (c) glycerol-lactate (3% w/v of glycerol, 2% w/v of 40% lactate) in M9 salts. Plating was done to ensure that each plate did not have more than 15 colonies, to avoid crowding effects. After 30 h of incubation at 37 °C, a single colony per media was carefully picked up by pipetting using small quantities (10–50 μl) of water and suspended in 2 ml LB. Serial dilution and plating was done to determine the CFU. The above steps were repeated after 76 h of incubation to determine the CFU of the colony that has reached saturation.

## Supplementary Information

Supplementary Information.

## Data Availability

The codes used in this study are provided in the manuscript Supplement.
