# Supplementary material for: Selection in a growing colony biases results of mutation accumulation experiments
Source: Sci Rep. 2022 Sep 14;12:15470. doi: 10.1038/s41598-022-19928-5 (PMC9475022; doi:10.1038/s41598-022-19928-5)
Supplement: Supplementary file 1 — Supplementary Information. [file 41598_2022_19928_MOESM1_ESM.docx]

**Supplement:**

**Selection in a growing colony biases results of mutation accumulation experiments.**

**Supplement Figures.**

**
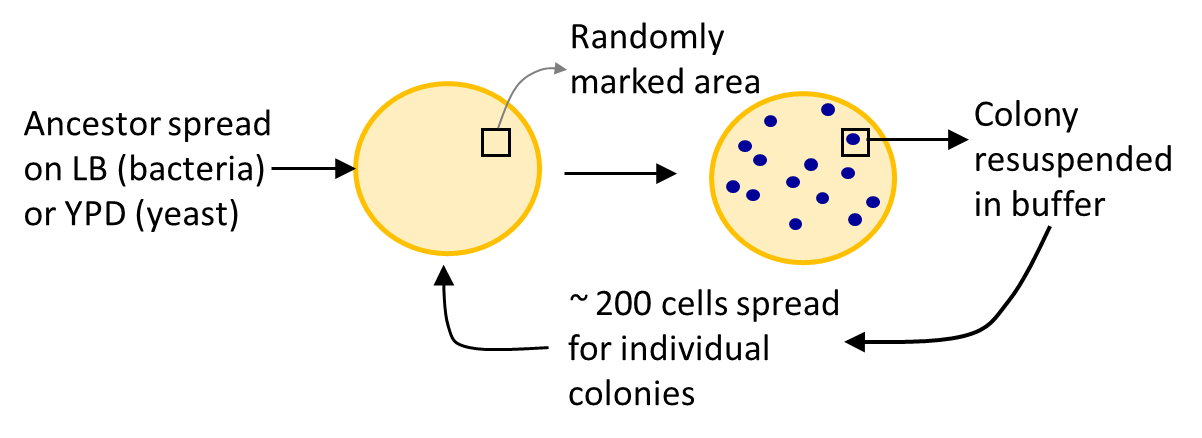
**

**Figure S1.** MA experiment methodology for yeast and bacteria. An area of the plate is marked and ~100 cells spread on the plate. A colony in the marked area is picked and the process repeated.

**
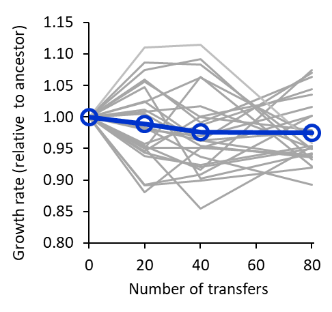
**

**Figure S2. Change in growth rate in a mutation accumulation (MA) experiment.** Twenty-two lines of *E. coli* were propagated in an MA experiment. Growth rate of the lines was recorded (grey lines) every 20 transfers and is represented as relative to that of the respective ancestor. The mean of the 22 lines is represented as dark blue curve. All measurements are averages of three repeats. The standard deviation in the growth rate measurement is less than 0.03%.

**
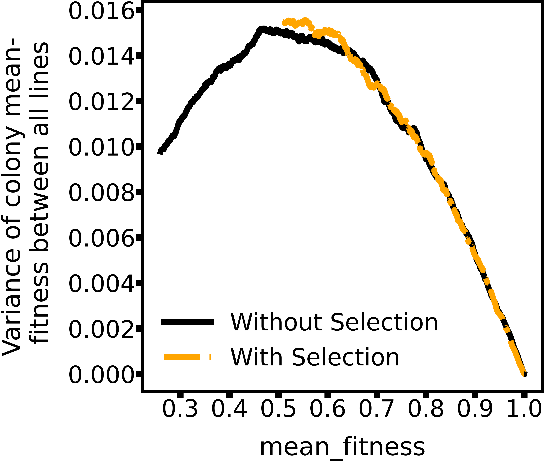
**

**Figure S3.** Starting with a mean fitness of 1.0, as the MA experiment proceeds, the inter-lines fitness variance increases. After a certain fitness (~0.4 in our simulations) however, the variance decreases rapidly, as all lines approach a small fitness value. In the “with selection” lines, the approach to fitness value of ~0.4 takes longer as compared to “without selection”, and hence, the “with selection” lines exhibit a higher variance after a certain number of transfers (as shown in **Figure 1G**). The data above is average of 1000 independent lines for each of the two conditions.

**
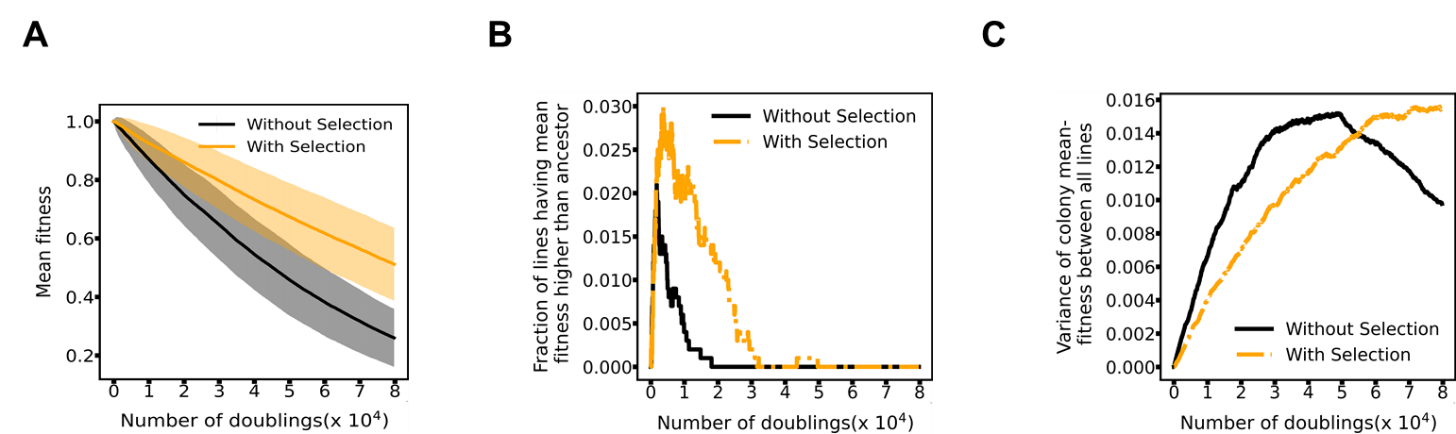
**

**Figure S4. (A)** Mean fitness (solid line) in an MA experiment (for 2000 transfers) and the standard deviation between the fitness of individual lines (shaded region). (Obtained p-values <0.05 by applying unpaired t-test before each transfer point)  **(B)** Fraction of the lines with fitness greater than that of the ancestor, as a function of number of transfers. **(C)** Fitness variance between lines as a function of number of transfers. (p-values <0.05, Levene test before each transfer point) All simulations were performed for *K* 6x10^8^, colony size 10^8^, and *b* 0.05.


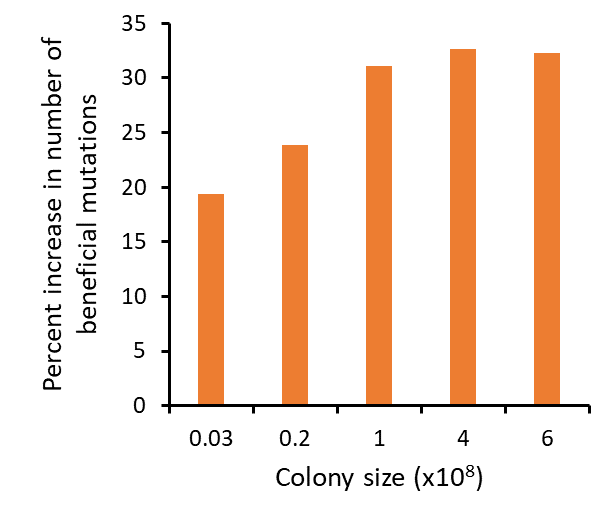


**Figure S5.** Relative increase in the beneficial mutations, compared to the control (“without selection”), as a function of colony size. In this simulation *K* equals 6x10^8^, and *b* equals 0.05. The data is average of 1000 independent runs. Y-axis is calculated as the following, [(# beneficial mutations in “with selection”)/(# beneficial mutations in “without selection”) – 1].

**A**

**
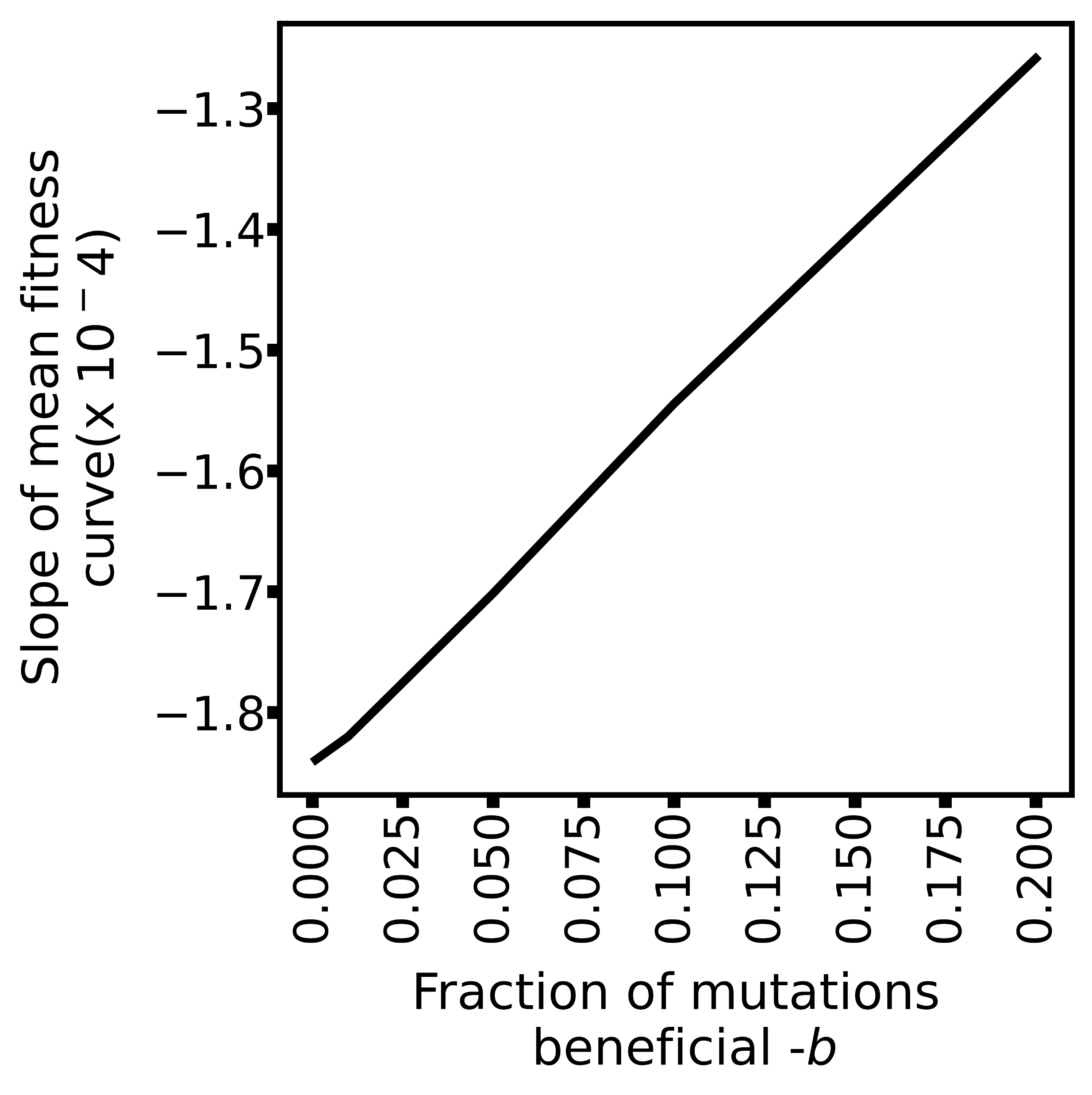
**

**B C**

**
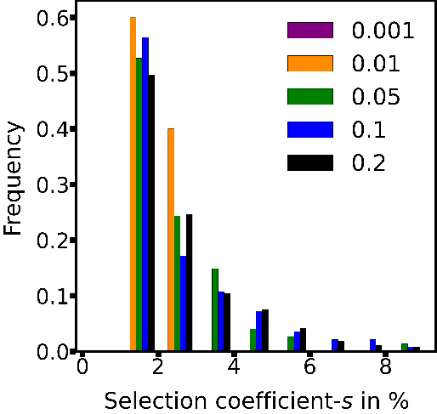

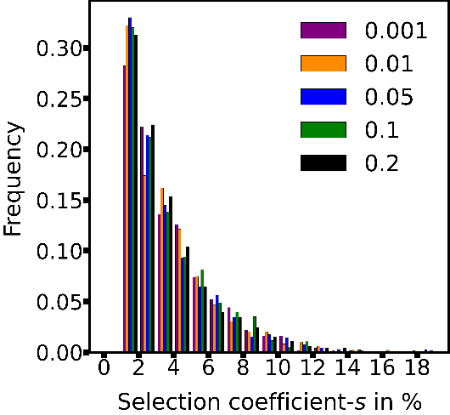
**

**D**

**
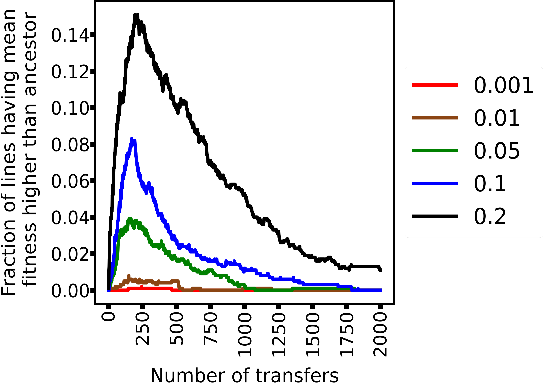
**

**Figure S6. (A) Slope of the fitness trajectory with the number of transfers increases with increasing *b*.** In the simulations, *K* equals 6x10^8^ and colony size is 10^8^. The slope is computed as an average of 1000 independent runs. Distribution of **(B)** beneficial and **(C)** deleterious mutations from the MA experiment simulation. (p-value for any pairwise distribution > 0.2; Kolmogorov-Smirnov Test) **(D)** Fraction of lines with increased fitness increases with *b.*

**A**


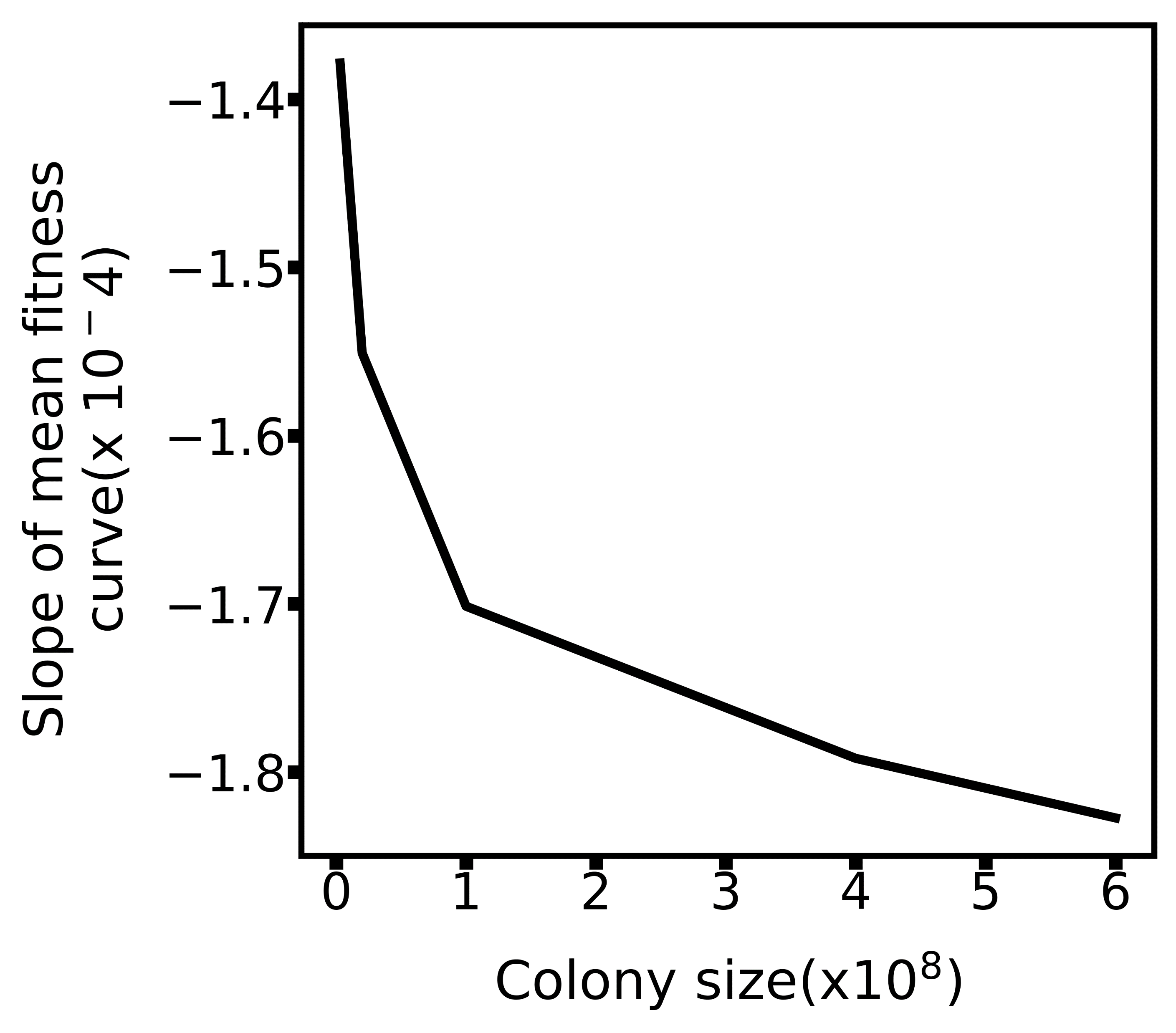


**B C**

**
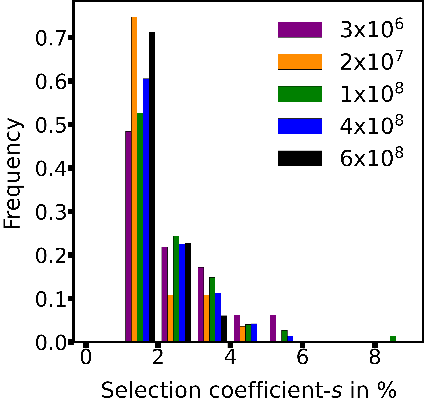

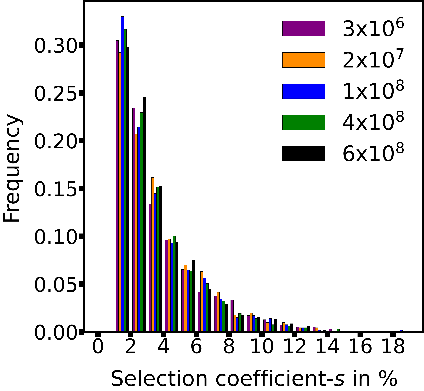
**

**D**

**
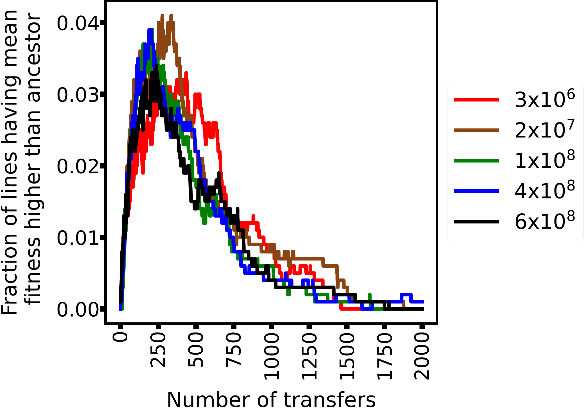
**

**Figure S7. (A) Slope of the fitness trajectory with the number of transfers increases with increasing colony size.** In the simulations, *K* equals 6x10^8^ and *b* is 0.05. The slope is computed as an average of 1000 independent runs. Distribution of **(B)** beneficial and **(C)** deleterious mutations from the MA experiment simulations (p-value for any pairwise distribution > 0.17; Kolmogorov-Smirnov Test). **(D)** Fraction of lines with increased fitness as a function of colony size at the time of transfer*.*


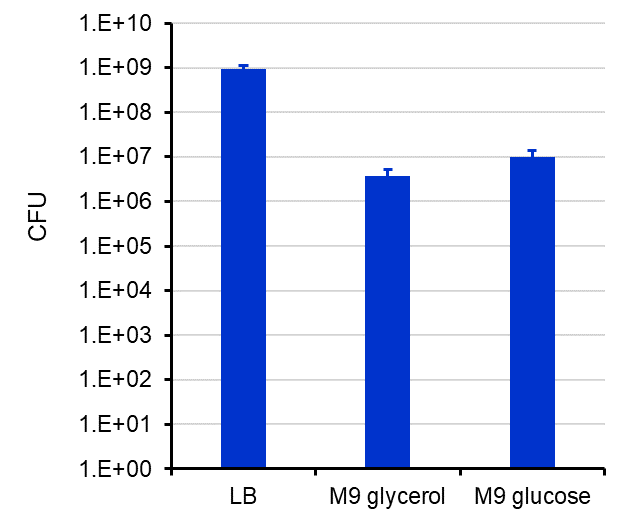


**Figure S7.** CFU in a colony after 30 h of growth on plates with different media compositions. All experiments were performed three independent times. The average and standard deviation is reported.

**A**


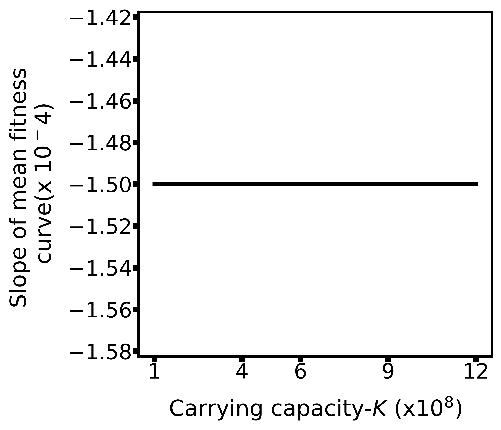


**B C**


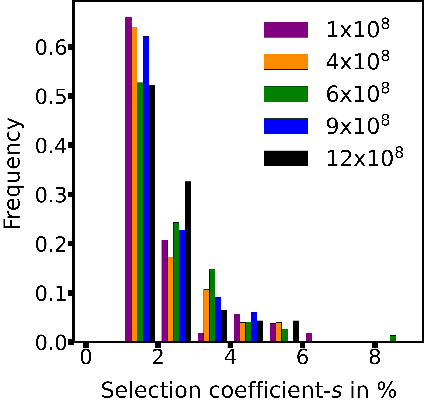

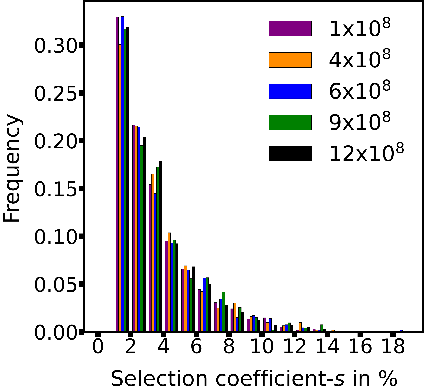


**D**


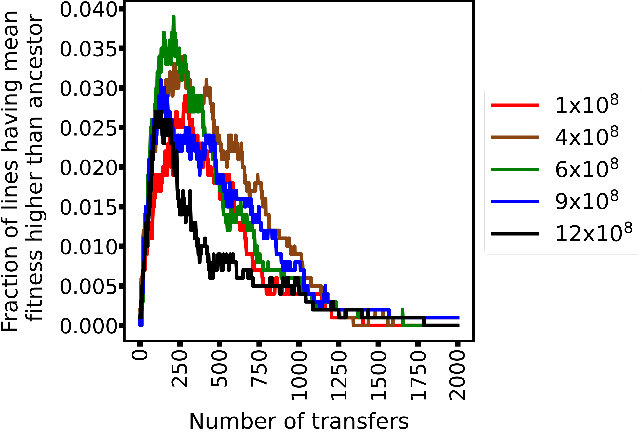


**Figure S9. (A) Slope of the fitness trajectory with the number of transfers does not change with increasing *K*, if transfer is done at small colony size.** In the simulations, colony size equals 10^8^ and *b* is 0.05. The slope is computed as an average of 1000 independent runs. Distribution of **(B)** beneficial and **(C)** deleterious mutations from the MA experiment simulation (p-value for any pairwise distribution > 0.62; Kolmogorov-Smirnov Test). **(D)** Fraction of lines with increased fitness as a function of *K.*

**A**


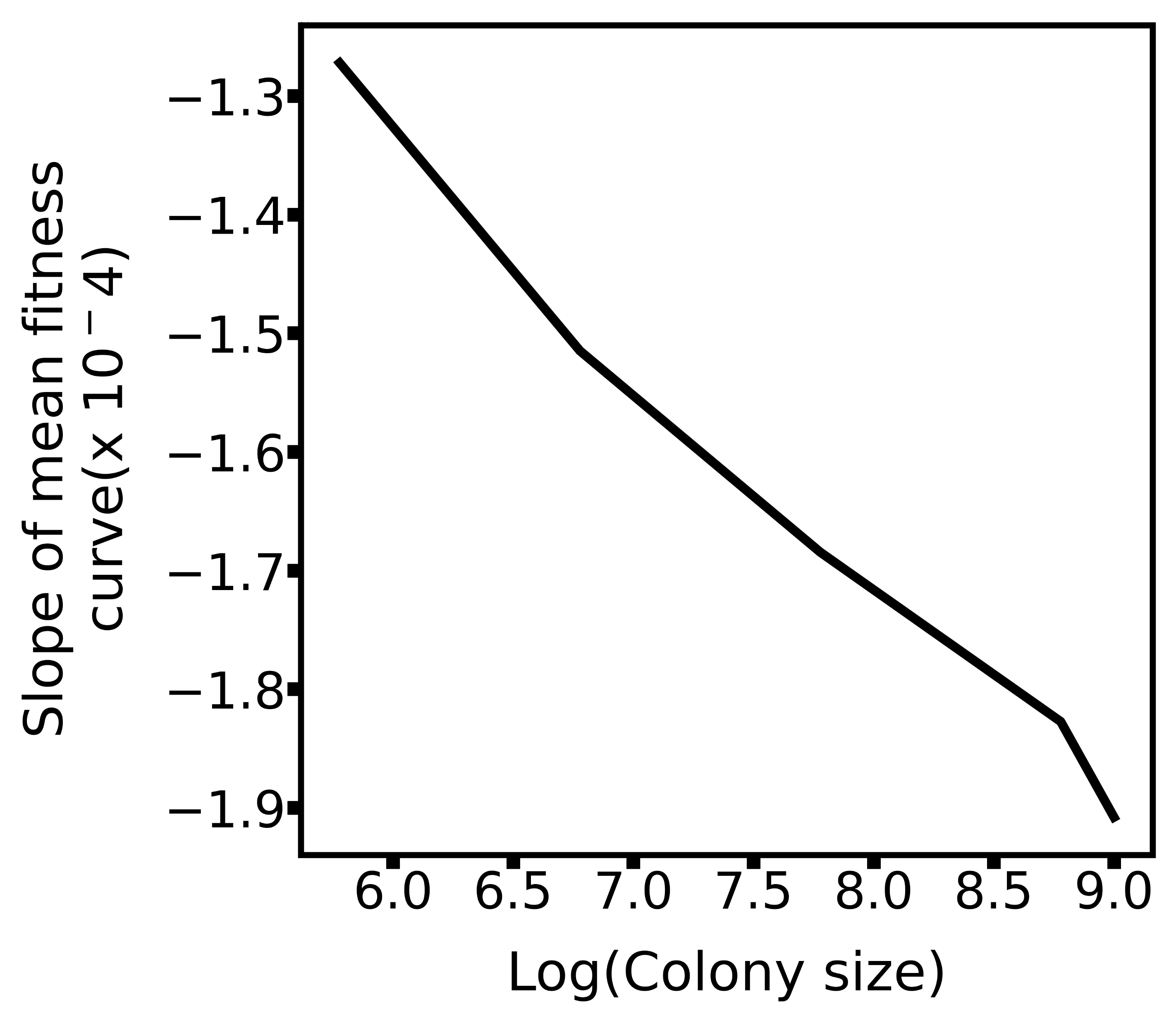


**B C**

**
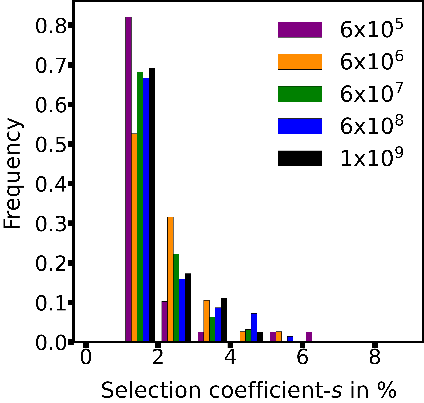

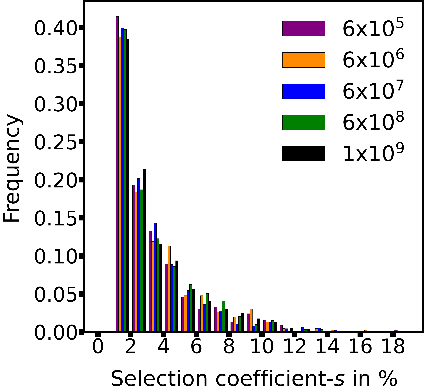
**

**D**


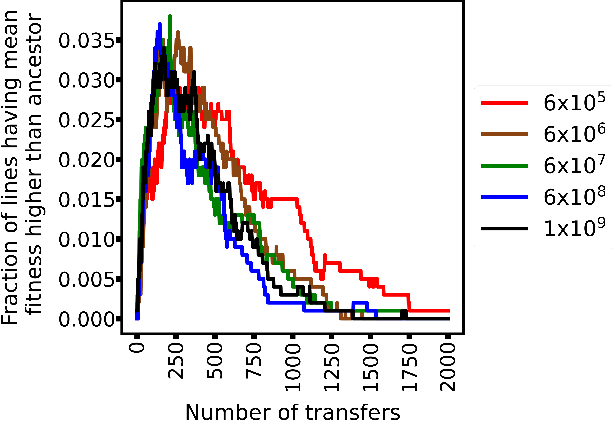


**Figure S10. (A) Slope of the fitness trajectory with the number of transfers when colony size equals carrying capacity *K*.** In the simulations, *b* is 0.05, and *K* equals colony size. The slope is computed as an average of 1000 independent runs. Distribution of **(B)** beneficial and **(C)** deleterious mutations from the MA experiment simulation (p-value for any pairwise distribution > 0. 21; Kolmogorov-Smirnov Test). **(D)** Fraction of lines with increased fitness as a function of *K* (in this case colony size at the time of transfer equals *K*)*.*

**A B**


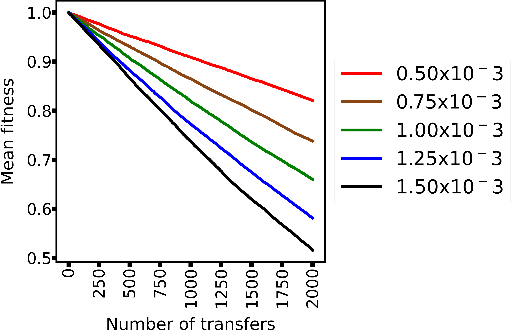

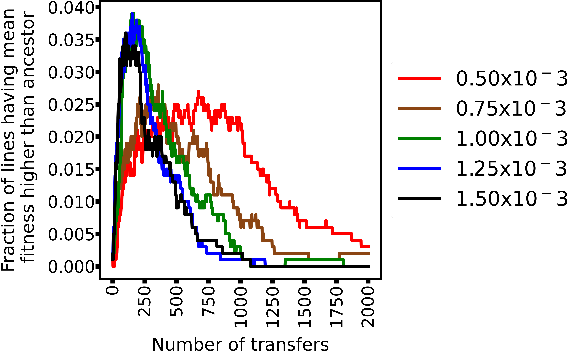


**Figure S11. Mutation rates affect the result of MA experiment.** **(A)** Fitness trajectory of an MA experiment with different mutation rates (per cell per generation). **(B)** Fraction of lines which exhibit fitness greater than the ancestral fitness is dictated by mutation rates. The simulations were performed for *K* equal to 6x10^8^ and colony size 10^8^. All results are an average of 1000 independent runs.

**
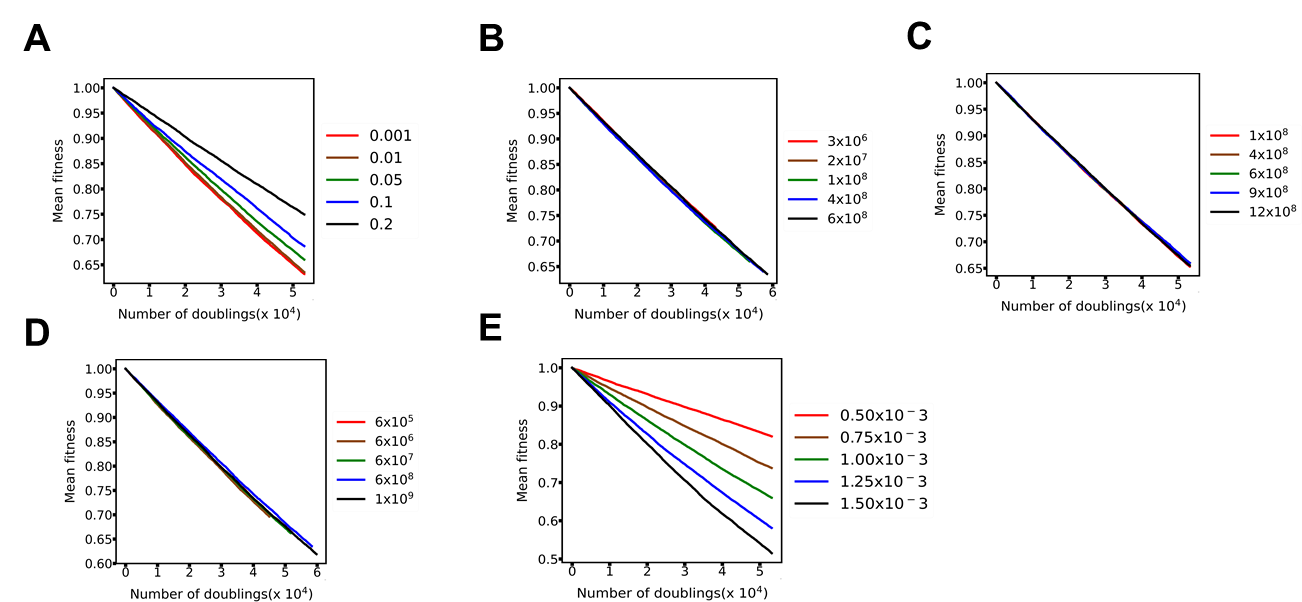
**

**Figure S12. (A)** Mean fitness of MA lines as a function of number of doublings. As the fraction of mutations that are beneficial increases, the fitness increases. **(B)** Mean fitness of MA lines as a function of number of doublings. The rate of decrease of fitness is independent of the size of colony at which the transfer is done. **(C)** Mean fitness of MA lines as a function of number of doublings. The rate of decrease of fitness is independent of the carrying capacity of the colony environment. **(D)** Mean fitness of MA lines as a function of number of doublings. The rate of decrease of fitness is independent of the size of colony at which the transfer is done (in this case *K* equals the colony size at which transfer was done). **(E)** Mean fitness of MA lines as a function of number of doublings. The rate of decrease of fitness is a function of the mutation rate of the organism.

**A B C**


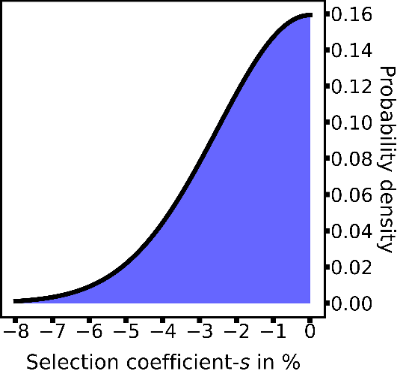

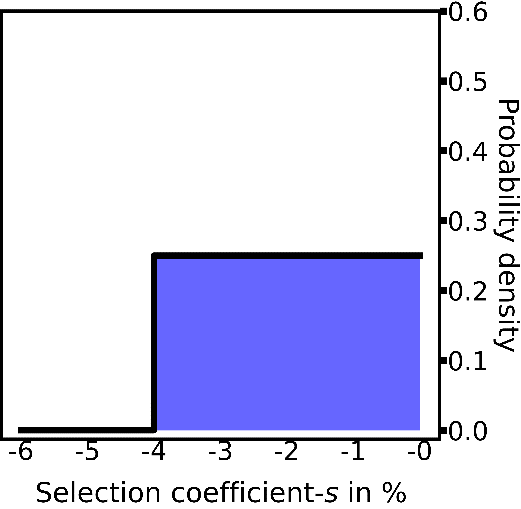

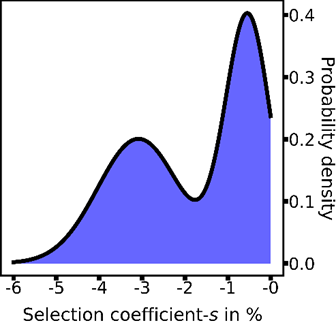


**D**


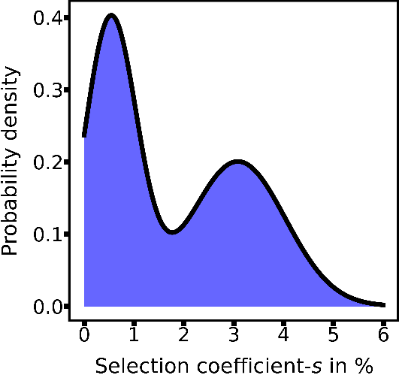


**Figure S13.** Alternate probability densities used for deleterious **(A-C)** and beneficial **(D)** mutations. **(A)** normal distribution, **(B)** uniform distribution, **(C)** and (**D)** mixture of normal distributions.


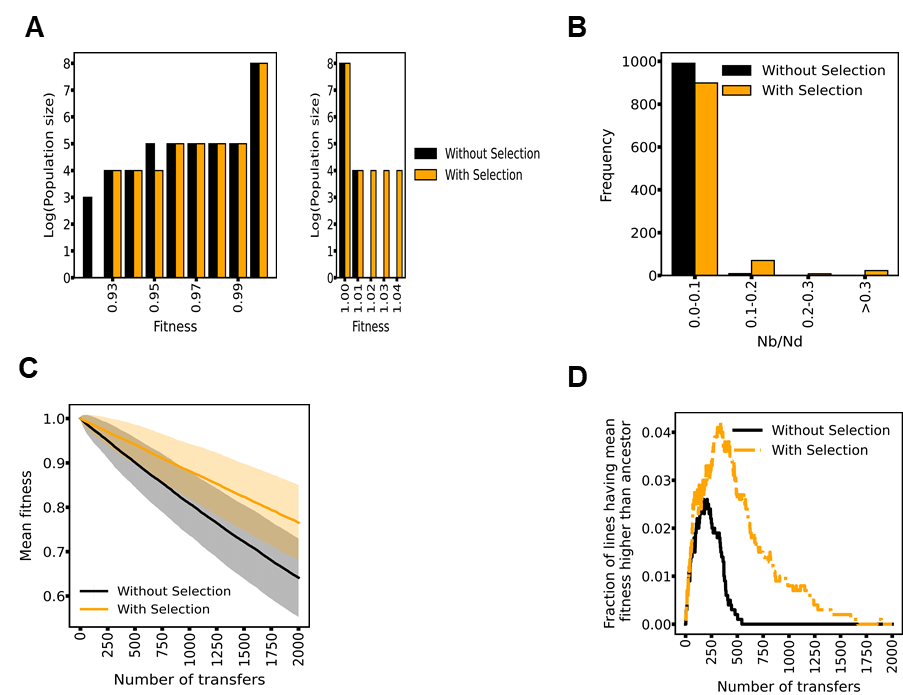


**Figure S14.** Colony growth and MA fitness trajectories when distribution effects of deleterious mutations are represented by a normal distribution. **(A)** Distribution of deleterious (left) and beneficial (right) mutants in one colony growth. (p < 0.16 (left: p-value 0.99, right: p-value 0.03), Kolmogorov-Smirnov test) **(B)** distribution of N_b_/N_d_ in 1000 colonies (p < 10^-07^, Kolmogorov-Smirnov test). **(C)** Average fitness trajectory of 1000 MA lines (shaded region is standard deviation). (Obtained p-values <0.05 by applying unpaired t-test at population before each transfer point) **(D)** Fraction of lines which exhibit increased fitness as compared to the ancestor.

**
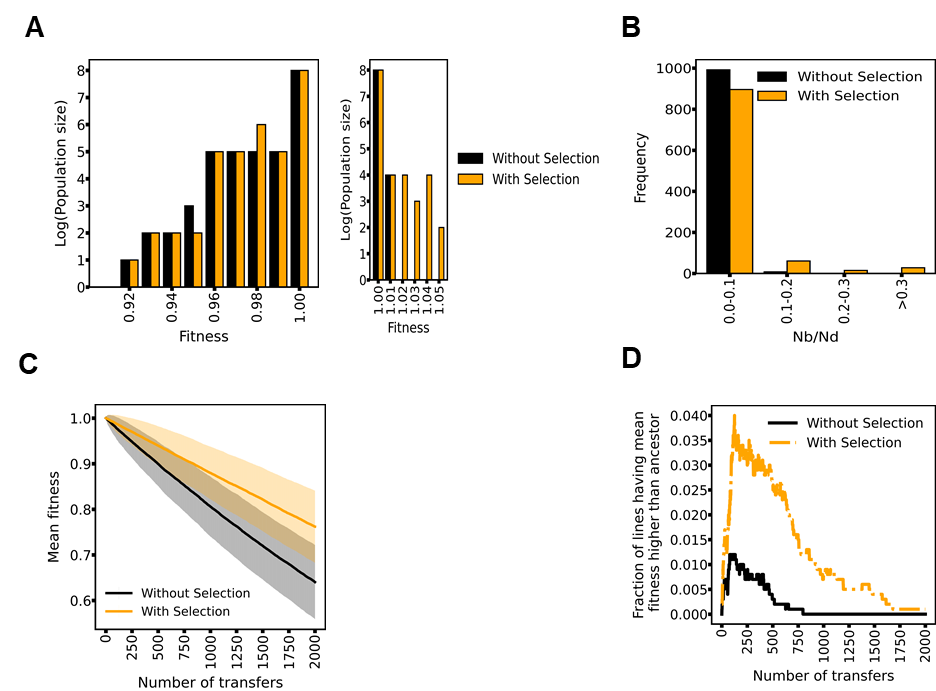
**

**Figure S15.** Colony growth and MA fitness trajectories when distribution effects of deleterious mutations are represented by a uniform distribution. **(A)** Distribution of deleterious (left) and beneficial (right) mutants in one colony growth (p < 0.1 (left: pvalue 1, right: p-value 0.01), Kolmogorov-Smirnov test). **(B)** Distribution of N_b_/N_d_ in 1000 colonies(p < 10^-07^, Kolmogorov-Smirnov test). **(C)** Average fitness trajectory of 1000 MA lines (shaded region is standard deviation) (Obtained p-values <0.05 by applying unpaired t-test at population before each transfer point), **(D)** Fraction of lines which exhibit increased fitness as compared to the ancestor.

**
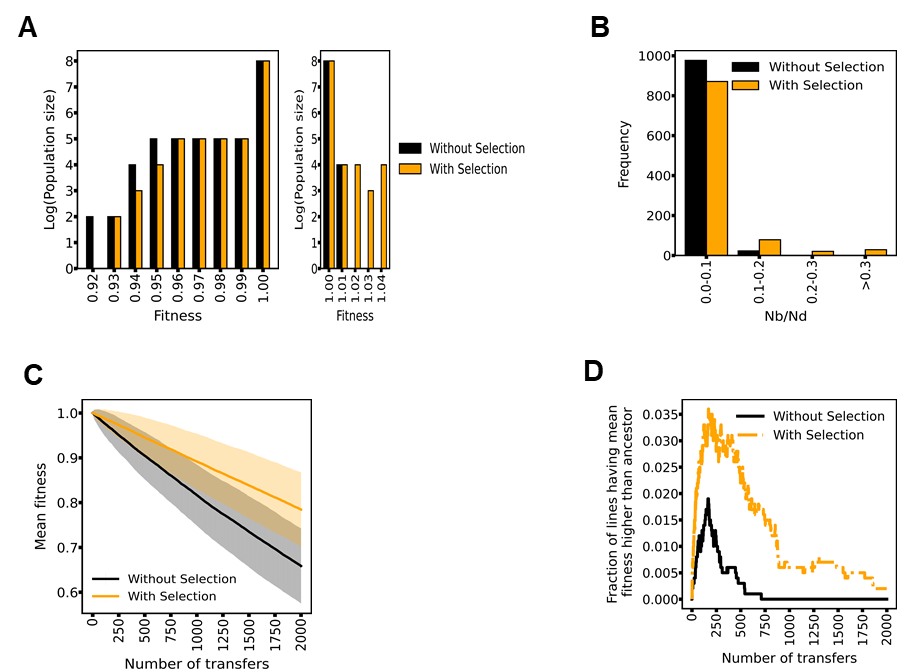
**

**Figure S16.** Colony growth and MA fitness trajectories when distribution effects of deleterious mutations are represented by a mixture of normal distributions. **(A)** Distribution of deleterious (left) and beneficial (right) mutants in one colony growth (p < 0.2 (left: p-value 0.99, right: p-value 0.04), Kolmogorov-Smirnov test). **(B)** Distribution of N_b_/N_d_ in 1000 colonies (p < 10^-08^, Kolmogorov-Smirnov test). **(C)** Average fitness trajectory of 1000 MA lines (shaded region is standard deviation) (Obtained p-values <0.05 by applying unpaired t-test at population before each transfer point), **(D)** Fraction of lines which exhibit increased fitness as compared to the ancestor.


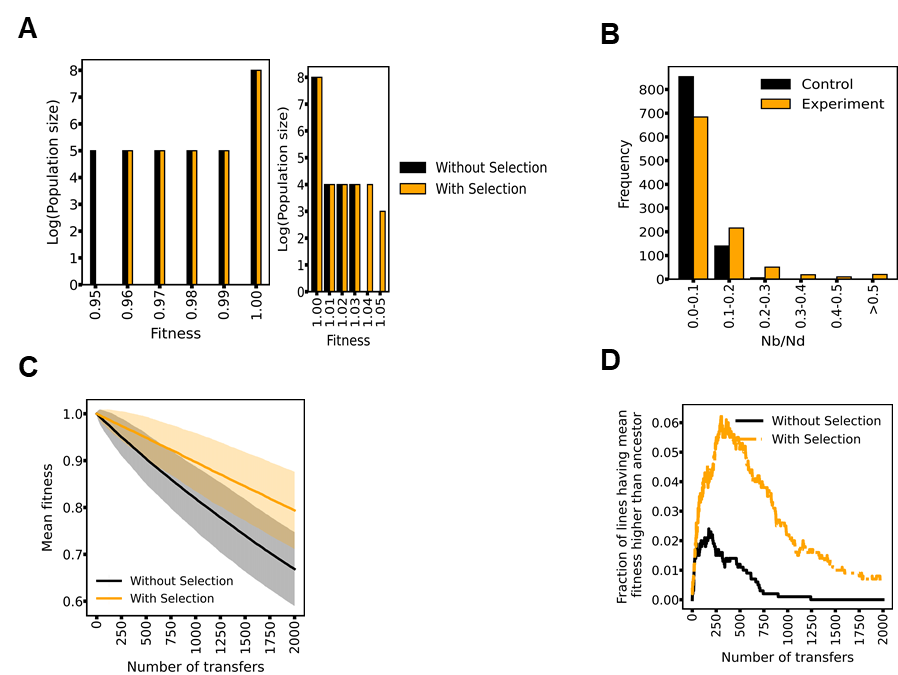


**Figure S17.** Colony growth and MA fitness trajectories when distribution effects of beneficial and deleterious mutations are each represented by mixtures of normal distributions. **(A)** Distribution of deleterious (left) and beneficial (right) mutants in one colony growth (p < 0.19 (left: p-value 0.8, right: p-value 0.3), Kolmogorov-Smirnov test). **(B)** Distribution of N_b_/N_d_ in 1000 colonies (p < 10^-08^, Kolmogorov-Smirnov test). (C) Average fitness trajectory of 1000 MA lines (shaded region is standard deviation) (Obtained p-values <0.05 by applying unpaired t-test at population before each transfer point), (D) Fraction of lines which exhibit increased fitness as compared to the ancestor.


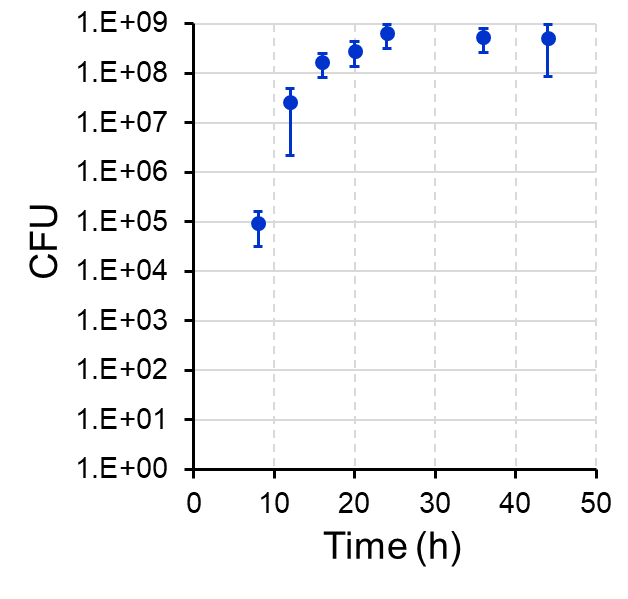


**Figure S18. CFU count in a growing *E. coli* colony with time.** The number of CFUs in a colony saturates at about 24 hours duration. All experiments were performed three independent times. In each run, three colonies were suspended and analysed for CFU. Average and standard deviation of the three experiments is reported.

**
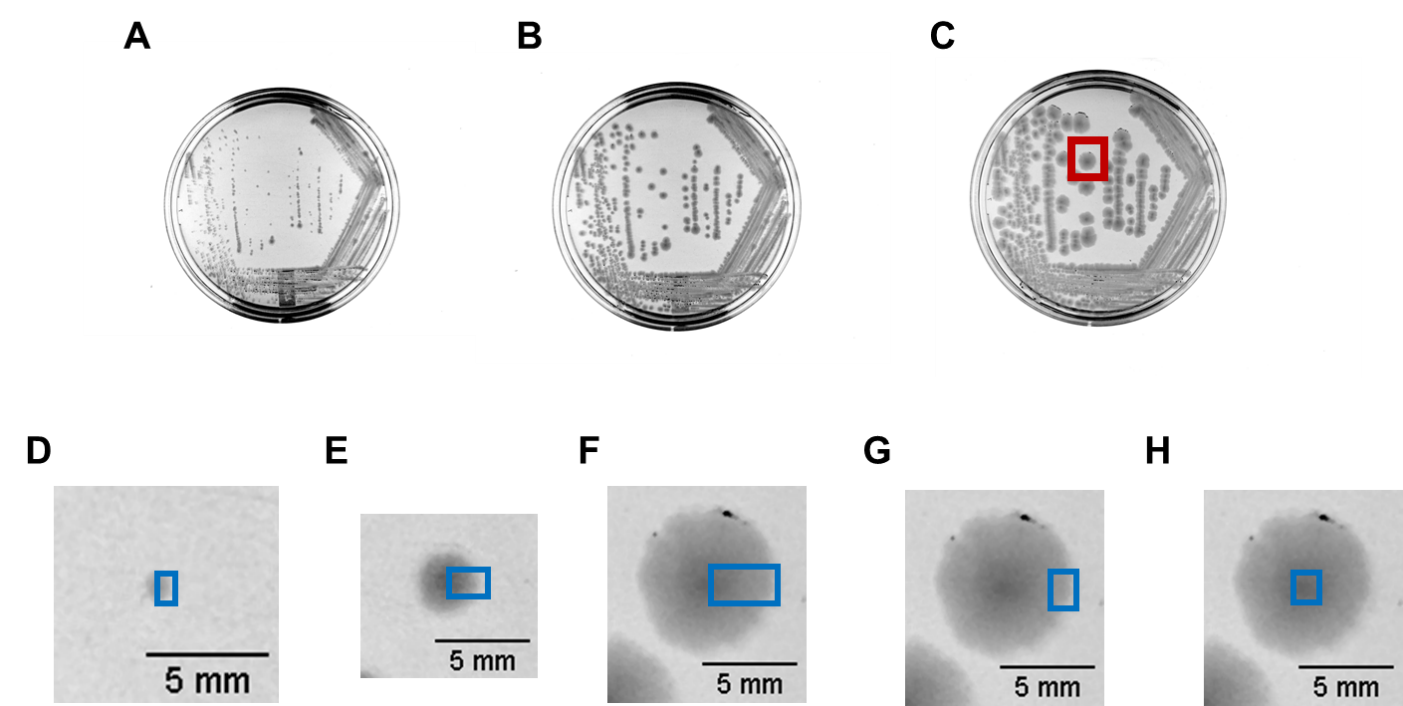
**

**Figure S19**. A representative figure for colony size growth on LB plates. *E. coli* colonies streaked on an LB plate at 37 deg C after **(A)** 8 hours, **(B)** 12 hours, and **(C)** 24 hours. For the five conditions in which MA experiments were performed, cells from within the area in blue boxes were picked up and spread on a fresh plate for single colony. (D) lines s1-s32, (E) lines m1-m32, (F) lines l1-l32, (G) lines e1-e32, and (H) lines c1-c32. The three image shown in (A-C) are for the same plate, and the images of the colony identified in red in Figure 3C at time 8 h (D) , 12 h (E), and 24 h (F-H) are shown for representation.

**Table S1.** List of parameters and their values in the simulation.

| **Parameter** | **Value** |
| --- | --- |
| Mutation rate, *μ* | 0.001 per cell per division |
| Fraction of mutations which are beneficial, *b* | 0.05 |
| Number of lines | 1000 |
| Number of transfers | 2000 |
| Exponential distribution parameter for beneficial mutations*,* mean - *λb* | 1 |
| Exponential distribution parameter (mean) for Deleterious mutations, mean- *λd* | 3 |
| Division time of the ancestor, *1/f0 (t0)* | 100 minutes |
| Half normal distribution parameter for Deleterious mutations, SD- *σ* | *σ =* 2.51 |
| Uniform distribution parameters for Deleterious mutations, minimum *– a1* and maximum – *a2* | *a1*=0. *a2*=4 |
| Mixture of normal distribution parameters: mean – *m,* Covariance- *Σ,* SD- *σ* | *m =1.75, Σ = 0.91, σ = 1.48* |

**Annexure I. Simulation codes.**

Simulating growth of a colony from one cell to a given colony size (with carrying capacity, *K*). This simulation follows two phases of growth, called the stochastic growth phase and the deterministic growth phase. A randomly picked cell is used to track growth, and simulate an MA experiment.

import random as rn

import pandas as pd

import numpy as np

from scipy.integrate import odeint, quad

import matplotlib.pyplot as plt

import math

########## Code ##########################################

# Mutation accumulation simulation

# Colony after stochastic growth -> Deterministic growth

##########################################################

##########################################################

# Convert fitness to time of division value

def cal_time_of_division(f,t0):

if f==0:

f= 1e-10

t= round((1/f*t0),2)

return t

# Create fitness bins

def create_fitness_bins(pop,lamda_b,lamda_d,f):

dbin = round((lamda_d/100),3)

bbin = round((lamda_b/100),3)

fitness =[]

population = [x[1] for x in pop]

lb = round(f-bbin,3)

while lb>=min(population):

fitness.append(lb)

lb = round(lb - bbin,3)

fitness = fitness[::-1]

fitness.append(f)

ub = round(f+bbin,3)

while ub<=max(population)+bbin:

fitness.append(ub)

ub = round(ub + bbin,3)

return fitness, bbin, dbin

# Binary search to search the correct bin to place the population according its mean fitness.

def binary_search(e,fitness,bbin):

mid = 0

start = 0

end = len(fitness)-1

step = 0

while (start <= end):

step = step+1

mid = (start + end) // 2

if e[1]>=fitness[mid]-round((bbin*0.5),3) and e[1]<fitness[mid]+round((bbin*0.5),3):

return mid

if e[1] < fitness[mid]-round((bbin*0.5),3):

end = mid - 1

else:

start = mid + 1

return -1

# Distribute the population based on their fitness

def distribute(pop,fitness,bbin):

length = len(fitness)

#print(fitness)

dist = [0]*length

for e in pop:

if e[1]<fitness[0]:

dist[0]+=e[0]

elif e[1]>=fitness[length-1]:

dist[length-1]+=e[0]

else:

i = binary_search(e,fitness,bbin)

dist[i]+=e[0]

return dist

# Growth function

def f(y, t, params,K):

N=y

derivs=[]

func = (1-sum(N)/K)

for i in range(len(y)):

derivs.append(params[i]*N[i]*func)

return derivs

# Growth function coupled ODE

def solve_ode(rate,K,IV,del_t,t0,t):

t = np.arange(0.0, t+1, del_t)

rate = [(x/t0) for x in rate]

#print(rate)

soln = odeint(f, IV, t, args=(rate,K))

return soln

# Calculate mean fitness of population

def cal_mean(population,fitness):

#print(population)

mean =0

total_p = 0

for i,j in zip(population,fitness):

mean+=(i*j)

total_p+=i

mean = round(mean/total_p,3)

return mean

# exponential distribution function

def exponential(x,lamda):

f = (1/lamda)*np.exp(-1*(1/lamda)*x)

return f

# probability calculation based onexponential distribution

def calculate_prob(fitness,lamda_d,lamda_b):

l =len(fitness)

#print(l)

I_d = [quad(exponential, 0, lamda_b*0.5, args=(lamda_d))[0]]

low = 0

up = lamda_b*0.5

for i in range(l):

low =up

up+=lamda_b

I_d.append(quad(exponential, low, up, args=(lamda_d))[0])

I_d.append(quad(exponential, up,np.inf, args=(lamda_d))[0])

I_b = [quad(exponential, 0, lamda_b*0.5, args=(lamda_b))[0]]

low = 0

up = lamda_b*0.5

for i in range(l):

low =up

up+=lamda_b

I_b.append(quad(exponential, low, up, args=(lamda_b))[0])

I_b.append(quad(exponential, up,np.inf, args=(lamda_b))[0])

return I_b,I_d

# adding new beneficial and deleterious mutations

def mutation_distribute(N,fitness,prob,pos,delet):

l =len(fitness)

Ans =[]

if delet:

for i in range(0,pos):

j = pos-i

Ans.append([round(N*prob[i]),fitness[j]])

Ans.append([round(N*sum(prob[pos:])),fitness[0]])

else:

for i in range(pos,l-1):

j = i-pos

Ans.append([round(N*prob[j]),fitness[i]])

Ans.append([round(N*sum(prob[l-pos-1:])),fitness[l-1]])

return Ans

# Simulate the Deterministic growth of the colony

def simulate_growth(population,t,f,mu,b_mu,lamda_b,lamda_d,K,size_check,control,t0,f0):

fitness_bins, bbin, dbin = create_fitness_bins(population,lamda_b,lamda_d,f)

distribution = distribute(population,fitness_bins,bbin)

probability_bins = calculate_prob(fitness_bins,lamda_d,lamda_b)

delta_t=1

dfe_size_check = []

mean_fit = f

while math.log10(size_check)-math.log10(sum(distribution))>0.01:

# Solving ODE to get populayion size in different bins after t0 units of time

if control:

N = solve_ode([f0]*len(fitness_bins),K,distribution,delta_t,t0,t)[int(t)]

else:

N = solve_ode(fitness_bins,K,distribution,delta_t,t0,t)[int(t)]

N_new = [(i-j) for i,j in zip(N,distribution)]

new_pop =[]

for i in range(len(N)):

new_pop.append([round((1-mu)*N_new[i])+distribution[i],fitness_bins[i]])

# Generating beneficial mutations

mut_dist = mutation_distribute(round(N_new[i]*mu*b_mu),fitness_bins,probability_bins[0],i,0)

new_pop.extend(mut_dist)

# Generating Deleterious mutations

mut_dist = mutation_distribute(round(N_new[i]*mu*(1-b_mu)),fitness_bins,probability_bins[1],i,1)

new_pop.extend(mut_dist)

distribution = distribute(new_pop,fitness_bins,bbin)

mean_fit = cal_mean(distribution, fitness_bins)

# Return distribution of final colony, mean fitness, fitness bins created and distribution (to check the first colony population distribution)

return distribution ,mean_fit,fitness_bins,distribution

################################ End of Code ################################################

import numpy as np

import stochastic as stoch

import deterministic as determ

########## Code ##############################################

# Mutation accumulation simulation

# Cell->stochastic growth -> Deterministic growth -> Colony

# -> pick a random cell -> Cell-> ....................

##############################################################

# Convert fitness to time of division value

def cal_time_of_division(f,t0):

if f==0:

f= 1e-10

t= round((1/f*t0),2)

return t

# binary search function

def binary_search(element,arr1,arr2):

mid = 0

start = 0

end = len(arr1)-1

step = 0

while (start <= end):

step = step+1

mid = (start + end) // 2

if element >sum(arr1[:mid]) and element<=sum(arr1[:mid+1]):

return arr2[mid]

if element <= sum(arr1[:mid]):

end = mid - 1

else:

start = mid + 1

return -1

# Selecting the cell for the trnasfer based on the probability calculated by its distribution in the fitness bins

def select_cell_for_transfer(pop_dist,t0,fitness):

prob = [[pop_dist.index(x),x/sum(pop_dist)] for x in pop_dist]

u = np.random.uniform(0,1)

prob = sorted(prob, key = lambda x: x[1])

prob_val = [x[1] for x in prob]

prob_index =[x[0] for x in prob]

l = len(fitness)

if u>sum(prob_val[:l-1]):

i = prob_index[l-1]

elif u<=prob_val[0]:

i = 0

else:

i = binary_search(u,prob_val,prob_index)

f = fitness[i]

t = cal_time_of_division(f,t0)

return t,f

## Single line of MA experiment simulation for 'n_transfers' number of transfers

def ma_simulation(N0,f0,t0,mu,b_mu,lamda_b,lamda_d,Size_check,K,n_transfers):

############ Control ################################################################################################

final_dist_control = []

mean_fitness_control=[]

fit_list_control =[]

founder_fit_control =[]

f=f0

for transfer in range(n_transfers+1): ## MA experiment after n_transfers number of tranferring

population_control = stoch.simulate_growth(t0,f,mu,b_mu,lamda_b,lamda_d,1,t0,f0)## Get the population state after Stochastic growth

new_pop,mean_fit,fitness,dfe = determ.simulate_growth(population_control,t0,f,mu,b_mu,lamda_b,lamda_d,K,Size_check,1,t0,f0)

if transfer==0:

DFE_control_1=dfe # distribution of population in first colony in one line

final_dist_control.append(new_pop)

fit_list_control.append(fitness)

mean_fitness_control.append(mean_fit)

t,f = select_cell_for_transfer(final_dist_control[transfer],t0,fitness)

founder_fit_control.append(f)

##################################################################################################################################

################################### Experiment ##################################################################################

final_dist = []

mean_fitness=[]

fit_list=[]

founder_fit =[]

t=t0

f=f0

for transfer in range(n_transfers+1): ## MA experiment after n_transfers number of tranferring

population = stoch.simulate_growth(t,f,mu,b_mu,lamda_b,lamda_d,0,t0,f0)## Get the population state after Stochastic growth

new_pop,mean_fit, fitness,dfe = determ.simulate_growth(population,t,f,mu,b_mu,lamda_b,lamda_d,K,Size_check,0,t0,f0)

if transfer==0:

DFE_1=dfe # distribution of population in first colony in one line

fit_list.append(fitness)

final_dist.append(new_pop)

mean_fitness.append(mean_fit)

t,f = select_cell_for_transfer(final_dist[transfer],t0,fitness)

founder_fit.append(f)

######################## Plotting results and observations ############################################################

#return mean fitness of colonies of all transfers, Distribution of population, fitness bins created, the fitness of founder cell in every transfer in both control and experiment

return mean_fitness_control, mean_fitness, DFE_control_1, DFE_1, fit_list_control[0], fit_list[0], founder_fit_control, founder_fit

############## End of the Code ###########################################################

import numpy as np

import math

import ma_simulation as ma

###### Plot the population distribution according to fitness bins

def plot_distribution(dist,fit_bins):

plot_data =[]

for i in range(len(dist)):

if dist[i]!=0:

plot_data.extend([fit_bins[i]]*round(math.log10(int(dist[i]))))

return plot_data

########## Find fraction of fitness higher than ancestor ###########

def find_fraction_high(mean_fitness,n,f0):

ans =[]

for i in range(mean_fitness.shape[1]):

e = np.where(mean_fitness[:,i]>f0)

ans.append(len(e[0])/float(n))

return ans

########## Calculate Nb/Nd value for fisrt colony at at size= colony size #################

def Cal_Nb_Nd(dist,fit,f0):

ans =[]

for i,j in zip(dist,fit):

nb = 0.0

nd =1e-6

for k,l in zip(i,j):

if l<f0:

nd+=k

elif l>f0:

nb+=k

ans.append(round(nb/nd,3))

return ans

#########################################################################

## N =1000 lines of experiments

N_lines = 1000

###################### Parameters ###################################

N0 = 1.0 # founder cell

f0 = 1.0 # fitness of founder cell

t0 = 100.0 ## 100 min - Time of division of founder cell

mu = 0.001 # mutation rate

b_mu = 0.05 # fraction of mutations which are beneficial

lamda_b = 1 # lamda_b is the mean of the exponential distribution of fitness effects of beneficial mutations

lamda_d = 3*lamda_b # lamda_d is the mean of the exponential distribution of fitness effects of deleterious mutations

Size_check = 1e8 #Colony size at which cell is transferred to next plate

K=6e8

n_transfers = 2000

## List of mean fitness of each line in control.

mean_fitness_C = []

## DFE of first colony at population size =colony size

DFE_control = []

## Fitness bins of first colony of control

fit_bins_control =[]

### fitness of founder cell in every transfer in control

found_fit_C=[]

## List of mean fitness of each line in experiment.

mean_fitness = []

## DFE of first colony at population size =colony size

DFE = []

## Fitness bins of first colony

fit_bins =[]

### fitness of founder cell in every transfe

found_fit =[]

for i in range(N_lines):

print("Simulation of line number:" + str(i+1))

# ma.ma_simulation() returns number of transfers in each line(n_transfers), mean fitness of each line both control and experiment(C/NC),

C, NC, dfe_C, dfe_NC, fit_c, fit_nc, found_c,found_nc = ma.ma_simulation(N0,f0,t0,mu,b_mu,lamda_b,lamda_d,Size_check,K,n_transfers)

mean_fitness_C.append(C)

mean_fitness.append(NC)

DFE_control.append(dfe_C)

DFE.append(dfe_NC)

fit_bins_control.append(fit_c)

fit_bins.append(fit_nc)

found_fit_C.append(found_c)

found_fit.append(found_nc)

mean_fitness_C = np.array(mean_fitness_C)

mean_fitness = np.array(mean_fitness)

founder_control =np.array(found_fit_C)

founder = np.array(found_fit)

############### Plotting fraction of lines having fitness higher than ancestor cell Vs number of transfers ################################

fract_control = find_fraction_high(mean_fitness_C,N_lines,f0)

fract = find_fraction_high(mean_fitness,N_lines,f0)

############### Plotting frequency distribution Nb/Nd of first colony of all 100 0lines. ################################

Nb_Nd_control = Cal_Nb_Nd(DFE_control,fit_bins_control,f0)

Nb_Nd = Cal_Nb_Nd(DFE,fit_bins,f0)

################################## End of the Code ##########################################

import random as rn

import pandas as pd

import numpy as np

import matplotlib.pyplot as plt

########## Code ##################

# Mutation accumulation simulation

# Stochastic growth of a cell into colony

##################################

# Convert fitness to time of division value

def cal_time_of_division(f,t0):

if f==0:

f= 1e-10

t= round((1/f*t0),3)

return t

# Update list of waiting time after deleting the min_waiting time for division

def update_list(ind, array,op):

if op:

array = [round(x-array[ind],3) for x in array]

return (array[:ind]+array[ind+1:])

# Get the final list of [population size, fitness] of different bacteria in population after stochastic step

def get_status(wt):

ans =[]

for i in wt:

ans.extend(i[:])

return ans

# Get total population size of bacteria

def sum_n(wt):

tot = 0

for i in wt:

tot+=i[0]

return tot

# Simulating the Stochastic growth phase

def simulate_growth(t,f,mu,b_mu,lamda_b,lamda_d,control,t0,f0):

# initital_genotype

waiting_time =[[[1,float(f)]]] # list of [1 founder cell, fitness of cell]

waiting_time_only = [float(t)] # list of [waiting time for division = time of division for first cell]

N_max =60000 # Approx max population size allowed in stochastic phase

tot=1 #initially only 1 bacteria present in environment

Flag = 1 # To check if required colony size is reached or not

while Flag:

# ----------------selecting min time for division------------------------

t_min = min(waiting_time_only)

index1 = waiting_time_only.index(t_min)

old_gen = waiting_time[index1] # Selecting the cell population with min waiting time for division

tot = sum_n(old_gen)

if tot>N_max/2:

Flag=0

# Updating waiting time list : After selecting the cells with min waiting time for division

waiting_time = update_list(index1,waiting_time,0)

waiting_time_only = update_list(index1,waiting_time_only,1)

# ------------------------------------------------------------------------

## ------------------------ New generation ----------------------------------

new_gen = [] ## New generation population

for bact in old_gen:

if control:

new_gen.append([bact[0],bact[1],t0])

else:

t_new = cal_time_of_division(bact[1],t0)

new_gen.append([bact[0],bact[1],t_new])

U= np.random.uniform(0,1,bact[0]) ## Generating uniform random numbers

for k in U:

if k>mu:

new_gen[old_gen.index(bact)][0]+=1

else:

m = np.random.uniform(0,1)

if m < b_mu:

# Generate 's' value from exponential distribution of beneficial mutations

s = np.random.exponential((lamda_b))

s1 = (bact[1]*s/100.0)

f_new = round((bact[1]+s1),3)

if f_new == bact[1]:

new_gen[old_gen.index(bact)][0]+=1

else:

if control:

new_gen.append([1,f_new,t0])

else:

t_new = cal_time_of_division(f_new,t0)

new_gen.append([1,f_new,t_new])

else:

# Generate 's' value from exponential distribution of deleterious mutations

s = np.random.exponential((lamda_d))

s1 = (bact[1]*s/100.0)

f_new = round((bact[1]-s1),3)

if f_new == bact[1]:

new_gen[old_gen.index(bact)][0]+=1

else:

t_new = cal_time_of_division(f_new,t0)

if control:

new_gen.append([1,f_new,t0])

else:

t_new = cal_time_of_division(f_new,t0)

new_gen.append([1,f_new,t_new])

## ------------------------------------------------------------------------------------------------------------

for new in new_gen:

if new[2] not in waiting_time_only:

waiting_time_only.append(new[2])

waiting_time.append([new[:2]])

else:

index = waiting_time_only.index(new[2])

waiting_time[index].append(new[:2])

if not Flag:

break

population = get_status(waiting_time)

return population

############################ End of the Code ################################################
